# MORC3, a novel MIWI2 association partner, as an epigenetic regulator of piRNA dependent transposon silencing in male germ cells

**DOI:** 10.1038/s41598-021-98940-7

**Published:** 2021-10-14

**Authors:** Kanako Kojima-Kita, Satomi Kuramochi-Miyagawa, Manabu Nakayama, Haruhiko Miyata, Steven E. Jacobsen, Masahito Ikawa, Haruhiko Koseki, Toru Nakano

**Affiliations:** 1 Department of Pathology, Medical School, Osaka University, Yamada-oka 2-2 Suita, Osaka, 565-0871 Japan; 2 Graduate School of Frontier Biosciences, Osaka University, Yamada-oka 2-2 Suita, Osaka, 565-0871 Japan; 3 Laboratory of Medical Omics Research, Department of Frontier Research and Development, Kazusa DNA Research Institute, Kisarazu, Chiba 292-0818 Japan; 4 Department of Experimental Genome Research, Research Institute for Microbial Diseases, Osaka University, Yamada-oka 3-1 Suita, Osaka, 565-0871 Japan; 5 Department of Molecular, Cell, and Developmental Biology, University of California, Los Angeles, CA 90095 USA; 6 Department of Biological Chemistry, University of California, Los Angeles, CA 90095 USA; 7 Department of Howard Hughes Medical Institute, University of California, Los Angeles, CA 90095 USA; 8 RIKEN Center for Integrative Medical Sciences, 1-7-22 Suehiro-cho, Tsurumi-ku, Yokohama, Kanagawa 230-0045 Japan

**Keywords:** Spermatogenesis, Epigenetics, Non-coding RNAs, Developmental biology, Molecular biology

## Abstract

The PIWI (P-element-induced wimpy testis)-interacting-RNA (piRNA) pathway plays a crucial role in the repression of TE (transposable element) expression via de novo DNA methylation in mouse embryonic male germ cells. Various proteins, including MIWI2 are involved in the process. TE silencing is ensured by piRNA-guided MIWI2 that recruits some effector proteins of the DNA methylation machinery to TE regions. However, the molecular mechanism underlying the methylation is complex and has not been fully elucidated. Here, we identified MORC3 as a novel associating partner of MIWI2 and also a nuclear effector of retrotransposon silencing via piRNA-dependent de novo DNA methylation in embryonic testis. Moreover, we show that MORC3 is important for transcription of piRNA precursors and subsequently affects piRNA production. Thus, we provide the first mechanistic insights into the role of this effector protein in the first stage of piRNA biogenesis in embryonic TE silencing mechanism.

## Introduction

TEs (transposable elements) constitute about 40% of the mammalian genome^1,2^. Although a few copies of TEs can move in the genome, most of them are incapable of transposing. Some genomic mutations caused by transposon activity may play an important role in genome evolution and contribute to promoting biological diversity. However, the expression of transposons is potentially harmful, because it can disrupt genomic stability and induce damage to germlines, resulting in infertility^3^. Among the TEs, LINE1 (long interspersed nuclear element-1) retrotransposons are the most abundant in the mammalian genome and thousands of LINE1 genes are postulated to be intact and active in the mouse^4^. Retrotransposons including LINE1 are usually silenced by DNA methylation and histone modifications of their promoter regions^5^.

PIWI (P-element-induced wimpy testis) was originally discovered as an essential protein for gametogenesis in *Drosophila*. PIWI family proteins, evolutionally conserved in diverse organisms from *Caenorhabditis elegans* to mammals, are required for germ cell development and piRNA (PIWI-interacting RNA) biogenesis^6,7^. Embryonic piRNAs are germ cell-specific small noncoding RNAs of 25–31 nucleotides that bind to PIWI proteins and contribute to the silencing of TEs in the germlines. Notably, they are mostly derived from retrotransposons^8–11^. In mouse gonocytes, MILI (PIWIL2) and MIWI2 (PIWIL4) are essential for spermatogenesis and play critical functions in piRNA biogenesis and de novo DNA methylation at the retrotransposon region, respectively^12–15^.

In addition to PIWI proteins, other proteins like the TDRD proteins (Tudor domain containing proteins)^16–19^, RNA helicases such as MVH^20^ and MOV10L1^21^, MITOPLD endonuclease^22^, and PNLDC1 exonuclease^23–25^ have been reported to be involved in the piRNA pathway. The embryonic mouse germline piRNA biogenesis can be divided into primary and secondary pathways. Primary piRNAs are generated from long single-stranded RNAs, which are transcribed from piRNA clusters in the genome^26^. First, long capped and polyadenylated precursors are transcribed^27^. These long piRNA precursor transcripts are then fragmented by MITOPLD to phased precursor piRNAs (pre-piRNAs) that carry uridine at 5′ end (1st U) in 5′ → 3′ direction^28–30^. After the pre-piRNAs bind to MILI, the 3′ ends of pre-piRNA are trimmed by PNLDC1. In the secondary piRNA biogenesis pathway, MILI bound piRNA catalyzes a target transcript with 10 nt complementarity to create the piRNA by its slicing activity and generates a new secondary piRNA with a prominent bias for carrying adenine at position 10 (10th A)^31^. After the secondary piRNA is loaded to MIWI2, the piRNA-bound MIWI2 can be transported into the nucleus and recruit some transcriptional silencing related factors to mediate the DNA methylation of the target retrotransposon loci^32,33^.

MORC (originally named from the phenotype of microrchidia) is a nuclear protein family highly conserved in prokaryotic and eukaryotic cells, and the proteins of this family play an important role in gene regulation in multiple organisms^34^. In *Arabidopsis*, AtMORC1 and AtMORC6 play a critical role in TE repression with heterochromatin formation at retrotransposon regions, and the process is independent of DNA methylation and repressive histone modification^35,36^. In mice, there are six members of the MORC family, namely, MORC1, MORC2a, MORC2b, MORC3, MORC4, and SmcHD1^37^. MORC1, an essential protein in spermatogenesis, has emerged as a novel factor in the silencing of retrotransposons (LINE1 and IAP (intracisternal A-particle)) in male germ cells^38,39^. MORC1 plays an epigenetic function and is essential for the establishment of DNA methylation marks at retrotransposon genes in a piRNA pathway-independent manner^40,41^. Meanwhile, MORC2b is essential for meiosis and fertility in both sexes. Loss of MORC2b leads to misexpression of genes involved in spermatogenesis but does not cause de-repression of retrotransposons in testes^42^. MORC3 conventional knockout mice die at birth or within a day thereafter for unknown reasons and MORC3 is concluded to be essential for postnatal development^2^.

Transposon silencing in mouse embryonic male germ cells is ensured by MIWI2, which is guided to the appropriate gene locus through base-pairing between piRNAs and nascent RNAs transcribed from genomic TE regions and recruits some effector proteins to the regions. However, the identity of all the PIWI related effector proteins in the piRNA pathway and the molecular mechanisms involved in this process is currently not known. In this study, we identified and characterized the MORC3 protein as an associating partner of MIWI2 in mouse embryonic testis. We showed that MORC3 is important for transcription of piRNA precursors in the first step of piRNA biogenesis and is required for de novo DNA methylation of a retrotransposon in a piRNA pathway-dependent manner.

## Results

### MORC3 as a component of the MIWI2 protein complex

To characterize the molecular mechanism of retrotransposon silencing via the piRNA pathway, we searched for the proteins that associate with MIWI2 by immunoprecipitation using a ZF-MIWI2 Tg (zinc finer fused MIWI2 transgene) mouse. We previously produced two kinds of Tg mice bearing the ZF proteins which target to the common regulatory region of type A LINE1 elements. One expressed Flag-tagged ZF-MIWI2 fusion protein and the other expressed only Flag-tagged ZF protein in testis^32^.

**Figure 1:**
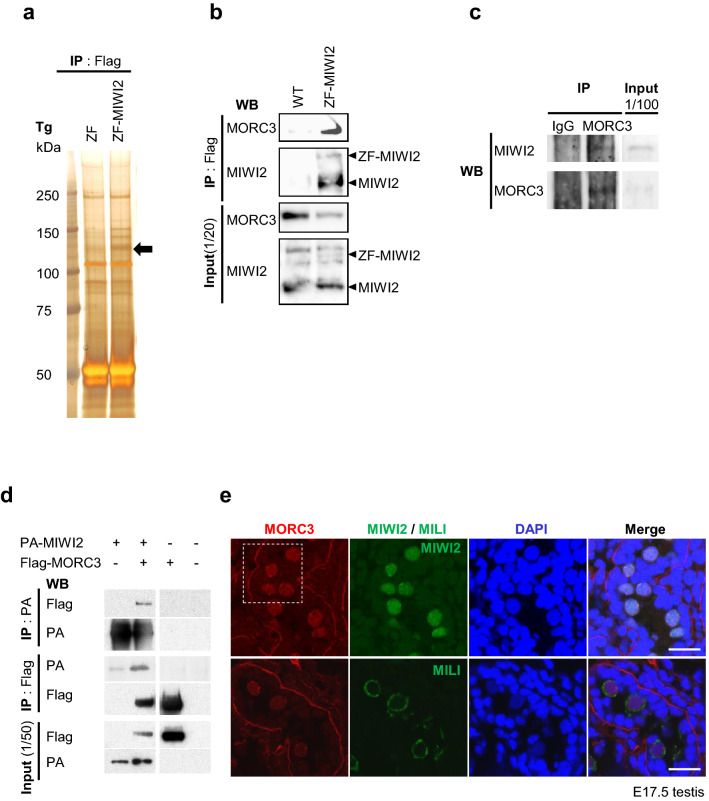
MORC3 as an interaction partner of MIWI2. (**a**) Silver staining of the postnatal testis proteins that co-immunoprecipitated with the anti-Flag antibody. Testis lysates of 21-day-old ZF only and ZF-MIWI2 Tg mice were subjected to IP (immunoprecipitation) with the anti-Flag antibody and SDS–PAGE was carried out. The 130 kDa protein that bound specifically to MIWI2 (black arrow) was examined by LC–MS/MS. Uncropped gel was shown in Supplementary Figure S1a. (**b**) Binding of ZF-MIWI2 to MORC3 in embryonic testes. IP of the embryonic testes lysates of E16.5 wild type and ZF-MIWI2 Tg mice was carried out with the anti-Flag antibody. The immunoprecipitated MORC3, ZF-MIWI2, and MIWI2 proteins were detected using the corresponding antibodies in western blotting. All samples were run on the same gel and transferred. Uncropped blots were shown in Supplementary Figure S1b. (**c**) Interaction of endogenous MIWI2 to MORC3 in embryonic testes. IP of the embryonic testes lysates of E16.5 wild type mouse was carried out with the anti-MORC3 antibody. The immunoprecipitated MIWI2 and MORC3 proteins were detected using the corresponding antibodies by western blotting. All samples were run on the same gel and transferred. Uncropped blots were shown in Supplementary Figure S1c. (**d**) In vitro co-IP assays for MIWI2 and MORC3. HEK 293T cells were transfected with the plasmids expressing PA-tagged MIWI2 and Flag-tagged MORC3. The 293T cells were co-transfected with the tagged protein expression constructs. The lysates were immunoprecipitated with the anti-PA or anti-Flag antibodies and subsequently subjected to western blotting with these antibodies. Each of IP : PA, IP : Flag, and input samples was run on the same gel and transferred. Uncropped blots were shown in Supplementary Figure S1d-i. (**e**) Co-immunostaining of the testes of the E17.5 wild type mice with the anti-MORC3 antibody (red), anti-MIWI2 (green) or anti-MILI (green) antibodies, and DAPI (blue) for DNA are shown. Scale bar, 20 μm. The enlarged images (inside of the white dotted line) are shown in Supplementary Figure S1l.

Immunoprecipitated proteins from the ZF-MIWI2 Tg testes of 21-day-old mice were subjected to SDS–PAGE (sodium dodecyl sulfate–polyacrylamide gel) electrophoresis and silver staining. Comparison of the ZF only and ZF-MIWI2 Tg testes samples showed that one band at around 130 kDa was present in the ZF-MIWI2 Tg but not in the ZF Tg testis samples. LC–MS/MS analysis revealed that the identity of the fragments of 26-peptide of the band was similar to those of the MORC3 protein (303/942 amino acids; 32% coverage) (Fig. 1a and supplementary Fig.S1a). Immunoprecipitation and western blotting analysis with the anti-Flag antibody using ZF-MIWI2 Tg testes confirmed the presence of MORC3 in the ZF-MIWI2 complex (Fig. 1b and supplementary Fig.S1b). Similarly, the interaction of endogenous MIWI2 and MORC3 was also observed as shown in Fig. 1c and supplementary Fig.S1c. To analyze whether the interaction between MIWI2 and MORC3 is direct, we overexpressed PA-tagged MIWI2 and Flag-tagged MORC3 in the HEK 293T human embryonic kidney cell line, in which no piRNA pathway proteins are expressed. As shown in Fig. 1d and supplementary Fig.S1d-i, the association between MIWI2 and MORC3 in the transfected 293T cells strongly suggested a direct interaction between them. Similar experiment with the overexpression of MILI showed that MILI was also capable of binding to MORC3 (supplementary Fig.S1j and k).

Next, by co-immunostaining, we examined the localization and the possible interaction of MORC3 with MIWI2 and MILI in embryonic testis. Both piP-body and pi-body are important organelles for piRNA production and contain MIWI2 and MILI, respectively. MORC3 was expressed together with MIWI2 in not only the nucleus but also in germinal granules, known as the piP-bodies. However, the cytoplasmic signal of MILI-containing granules or the pi-bodies was not merged with the staining signal of MORC3. Taken together, we conclude that MORC3 is a physiological interaction partner of MIWI2 but not of MILI in the embryonic testis (Fig. 1e and supplementary Fig.S1l).

### Subfertility associated with the loss of MORC3

To examine the function of MORC3 in male germ cells, we generated the MORC3 mutant mouse using the Cre-loxP system. *Tnap* (Tissue non-specific alkaline phosphatase)-Cre Tg mice, in which Cre is efficiently and specifically expressed in germ cells, have been frequently utilized^43,44^. We checked whether the allele was deleted by PCR (polymerase chain reaction) using the sorted male germ cells of the *Morc3* conditional homozygous and heterozygous mutant mice (Day 12) (supplementary Fig.S2a). The male germ cells were purified using the anti-EpCAM antibody, and the purification levels were confirmed by the high methylation status (> 90%) of the DMR (differentially methylated region) of a paternally imprinted gene, DMR-H19. As shown in supplementary Fig.S2b and S2c, loss of MORC3 expression was ensured both in the testis of E14.5, a period before the beginning of de novo DNA methylation, and the testis of 30-day-old mice (Day 30) by immunostaining.

**Figure 2:**
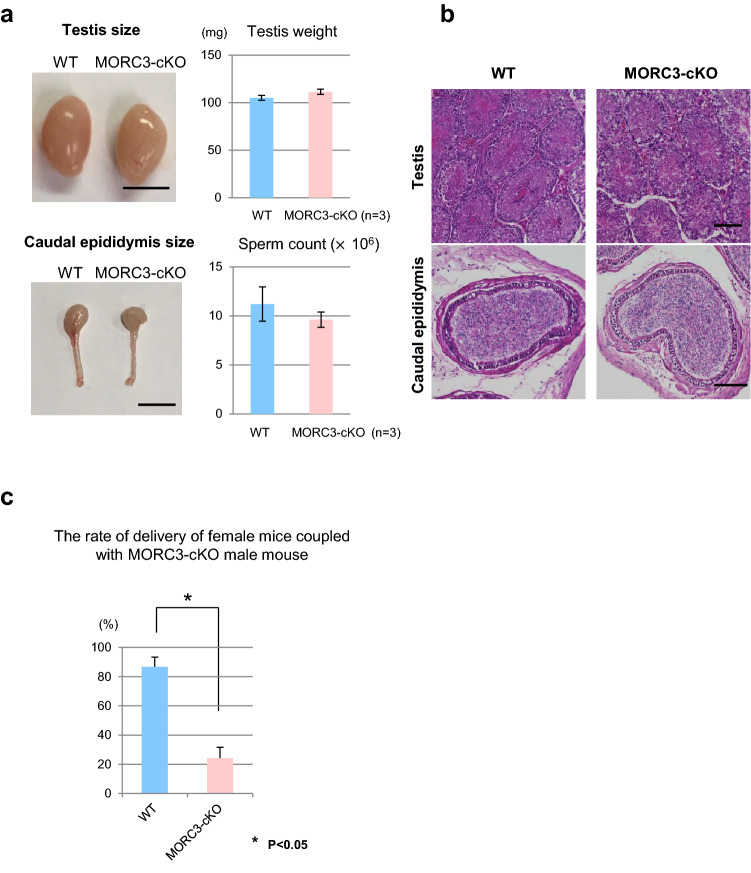
Subfertility of the MORC3 mutants. (**a**) Testes and caudal epididymis in the wild type and MORC3-cKO mice (left). Testis weights and sperm count in individual mice (right). Error bars denote SD. Scale bar, 5 mm. (**b**) HE staining of the testes and caudal epididymis of 8-week-old wild type and MORC3-cKO mice. Scale bar, 100 μm. (**c**) The percentage of delivery by female mice that mated with the MORC3-cKO male mice. Error bars denote SD. A significant difference (*p < 0.05 by the *t* test) between wild type and MORC3-cKO data is shown.

The size, weight, and HE staining of the testes of the MORC3 mutant adult mice were similar to those of wild type mice. In addition, the sperm numbers in the caudal epididymis were almost the same (Fig. 2a, b). The morphology and motility of the mutant sperm were also normal (supplementary Fig.S2d-f). However, the pregnancy rate of the female mice crossed with the mutant mice (25%) was significantly lower than that of female mice crossed with the wild mice (85%) (Fig. 2c and supplementary Fig.S2g). Although the litter size of the pregnant female mice was not significantly different (*p* = 0.08 by *t* test), the results indicated that the loss of MORC3 leads to subfertility of the male mice from the standpoint of pregnancy rate.

### De-repression of retrotransposons in the MORC3 mutant mice

**Figure 3:**
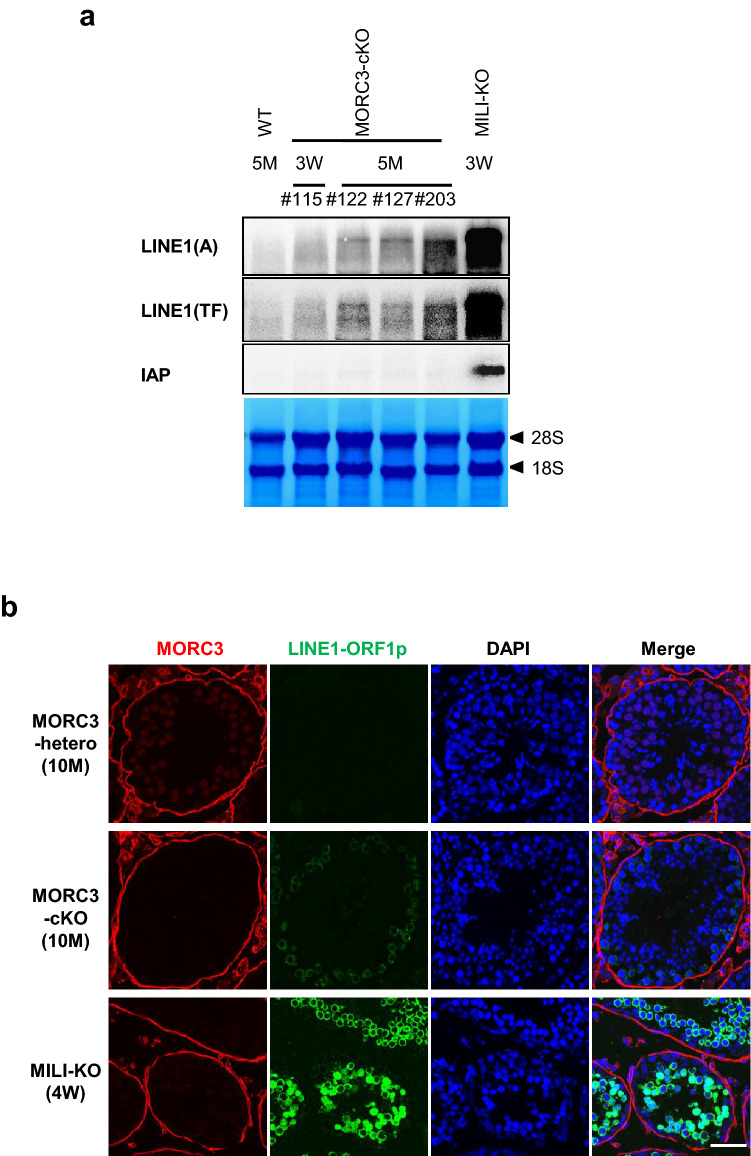

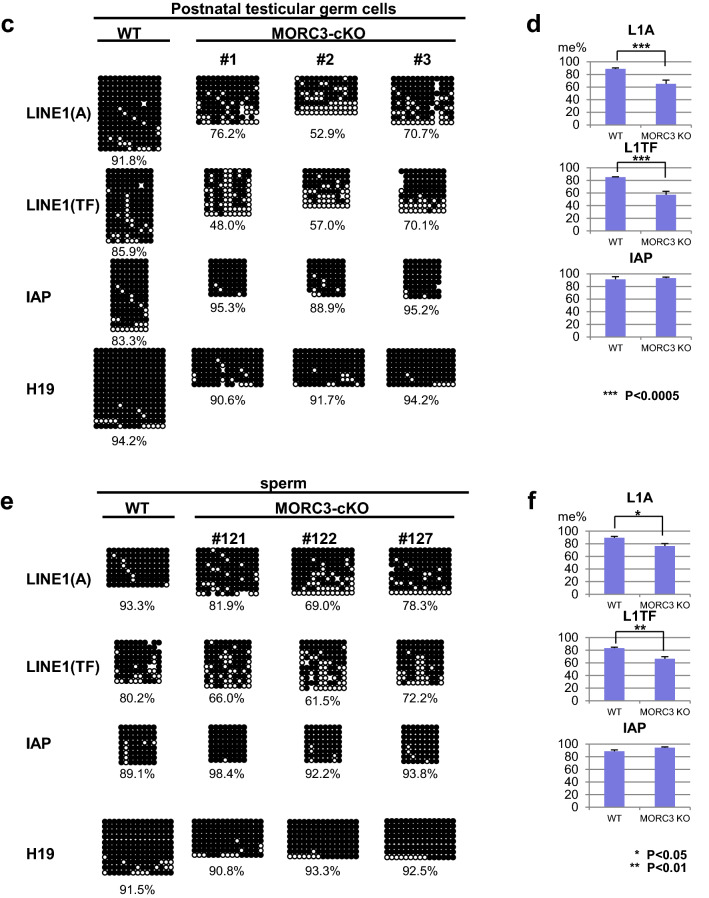
Expression of LINE1 retrotransposons and DNA methylation status of testicular male germ cells and sperm of the MORC3 mutants. (**a**) Northern blot analysis of LINE1 and IAP-1Δ1 retrotransposons in the testes of 5-month-old control wild type and MORC3-cKO mice, 3-week-old MORC3-cKO and MILI-KO mice. The 5′ UTRs of types A and TF LINE1 and the 3′ UTR of IAP were used as probes. All samples were divided equally and were run on 2 gels and transferred to 2 membranes. One is for detection of type A LINE1 and IAP. The other is for detection for type TF LINE1 and methylene blue staining. Uncropped blots were shown in Supplementary Figure S3a-d. (**b**) Co-immunostaining of the 10-month-old MORC3-heterozygous and the mutant mice and 4-week-old MILI-KO mouse testes with the anti-MORC3 antibody (red), anti-LINE1-ORF1p antibody (green), and DAPI (blue) for DNA are shown. Scale bar, 50 μm. (**c** and **e**) Representative data of bisulfite sequencing of 12-day-old male germ cells purified by the anti-EpCAM antibody (**b**) and sperm (**d**). The 5′ UTR region of LINE1 (types A [GenBank: M13002] and TF [GenBank: D84391]) and the LTR region of the 1Δ1-type IAP in chromosome 3qD were analyzed using the specific primers (sequences provided in the Supplementary information). Dots and circles represent methylated and unmethylated CpGs, respectively. Gaps in the methylation profiles represent mutated or unreadable CpG sites. The percentages of methylated CpGs are shown below each panel. (**d** and **f**) Statistical analyses of three independent bisulfite sequencing experiments using 12-day-old male germ cells (**c**) and sperm (**e**). Error bars denote SD. Significant differences (^*^p < 0.05, ^**^p < 0.01, ^***^p < 0.0005 by *u* test) between the indicated data of the type A and TF LINE1 (top and middle panels) are shown.

Based on the previous reports on the effect of the other MORC proteins on transposon silencing, we examined the expression of LINE1 and IAP genes in the testes of 5-month-old mice by northern blot analysis (Fig. 3a and supplementary Fig.S3a-d). LINE1 retrotransposons are divided based on their sequences into several distinct types, such as type A and type TF, both of which have similar and unique sequences in their coding and 5′ noncoding regions, respectively. The expression of type A and TF LINE1 transcripts were significantly upregulated, while that of IAP remained repressed in the MORC3 mutant testes. In contrast, the expression of LINE1 transcripts was lower in the MORC3 mutant mice than that of the MILI-null mouse, in which almost no piRNAs were produced. Such a de-repression of LINE1 transcripts was scarcely observed in the 3-week-old MORC3 mutant mouse. In addition, LINE1-ORF1p, a translational product from LINE1 transcripts could be detected in the 10-month-old MORC3 mutant testes but not in the embryonic testes by immunofluorescence analysis (Fig. 3b and supplementary Fig.S3e).

Next, DNA methylation levels of the LINE1 and IAP retrotransposons in the spermatocytes, before the pachytene stage, of the 12-day-old MORC3 mutant mice were examined by bisulfite sequencing (Fig. 3c, d). A significant reduction in the CpG methylation levels of type A and TF LINE1 genes was observed in the MORC3 mutant male germ cells, compared to the high DNA methylation of the control germ cells. Meanwhile, DNA methylation of the IAP gene was not impaired by MORC3 mutation and, correspondingly, no significant difference in IAP transcript levels was observed (Fig. 3a and supplementary Fig.S3a-d). The similarity in the phenotypes, i.e., low DNA methylation at LINE1 regions found in the MORC3 mutant, was observed in not only the postnatal testicular germ cells but also in the sperm (Fig. 3e, f). However, the degree of DNA methylation at LINE1 regions in the MORC3 mutant sperm was slightly higher than that in the 12-day-old MORC3 mutant male germ cells. We speculated that some germ cells that have quite low methylation levels at LINE1 regions would have been eliminated during the maturation process of spermatogenesis in the MORC3 mutant mice by mechanisms yet to be elucidated.

### Role of MORC3 in piRNA biogenesis

**Figure 4:**
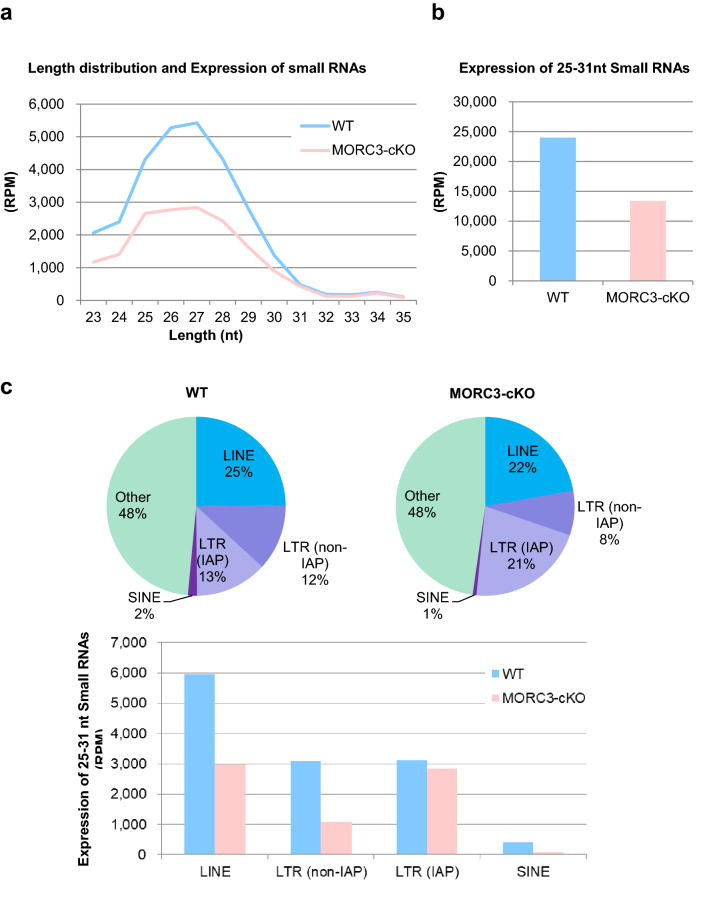

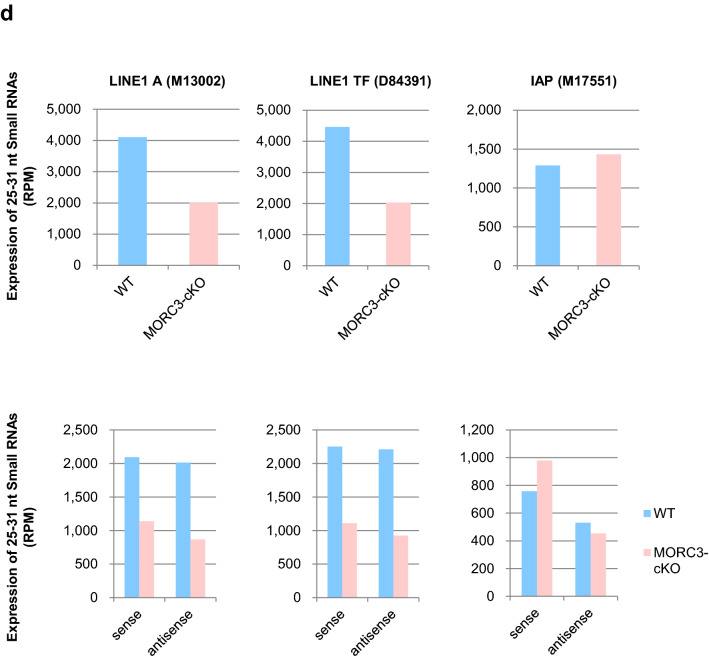

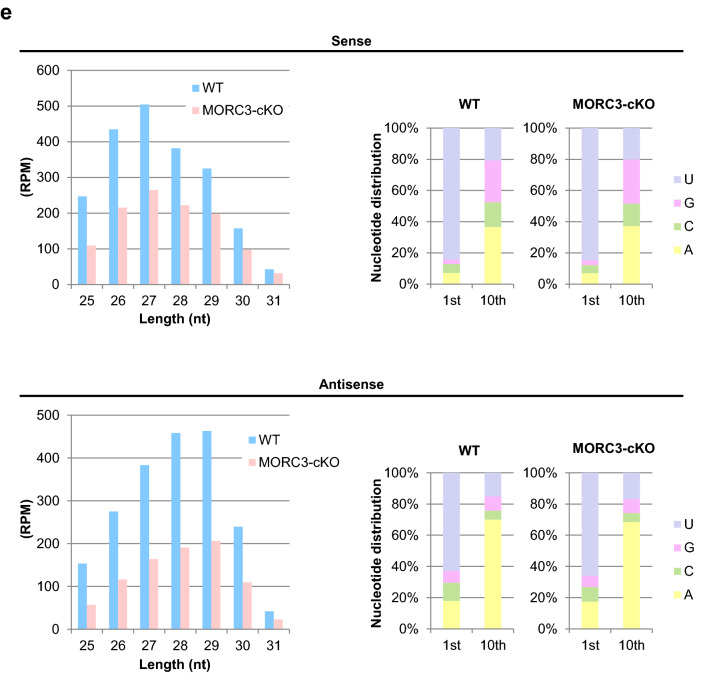
piRNA biogenesis and sequence profile in the MORC3 mutant embryonic testes. (**a**) Length distribution of small RNAs from E16.5 wild type and MORC3-cKO testes. Small RNAs were analyzed after ribosomal RNA (rRNA), micro RNA (miRNA), and transfer RNA (tRNA) mapped reads were removed by CLC Genomics Workbench. Light blue and pink bars show the wild type and MORC3-cKO data, respectively. The accession number of deep sequencing data is DRA011066. (**b**) Expression of 25–31 nt small RNAs corresponding to piRNAs in individual samples. (**c**) Genomic annotation and classification of repeat piRNAs based on retrotransposon class in the wild type and MORC3-cKO piRNA libraries (upper). Expression of piRNAs corresponding to LINE, LTR (non-IAP), LTR (IAP), and SINE in individual repeat piRNAs (bottom). (**d**) Expression of piRNAs derived from both strands (upper) and each strand (sense or antisense, bottom) corresponding to LINE1 A (M13002), LINE1 TF (D84391), and IAP (M17551) sequences in the wild type and MORC3-cKO piRNA libraries. (**e**) Length distribution of 25–31 nt small RNAs derived from each strand (sense or antisense, left). Nucleotide distribution of the 1st or 10th nucleotide of 25–31 nt small RNAs from each strand (right).

DNA methylation marks on LINE1 repeat elements are linked to the nuclear events of the piRNA-dependent DNA methylation pathway, in which MIWI2 is involved. To assess whether MORC3 plays any role in piRNA biogenesis, we purified the total small RNAs from wild type as well as the MORC3 mutant embryonic testes (E16.5) and subjected them to deep-sequencing. After trimming of miRNAs, rRNAs, and tRNAs from both total small RNA libraries using the CLC Genomics Workbench, the reads were mapped to the mouse genome (mm10, UCSC) and normalized to the read-depth of each library. Comparison of small RNA size distribution profiles among their libraries revealed that the number of 25–31 nt small RNAs (corresponding to the length of piRNAs) was significantly reduced in the MORC3 mutant testes (Fig. 4a, b). The expression of miRNAs, used as an internal control, was almost equal in them (supplementary Fig.S4a) and the genomic annotations for 25–31 nt small RNAs using the control and MORC3 mutant libraries indicated that much of reads were originated from retrotransposon-related sequences.

Analysis of piRNAs of the MORC3 mutant testes revealed that a significant decrease was largely contributed to the reduction of small RNAs derived from LINE elements (LINE), LTR elements except for IAP (LTR (non-IAP)), and SINE elements (SINE) (Fig. 4c). To evaluate the expression of piRNAs corresponding to LINE1 in detail, we mapped 25–31 nt small RNAs to type A and TF LINE1. While both sense and antisense piRNAs levels were decreased to about half, the proportion of 1st U sense and 10th A antisense piRNAs was not altered in the MORC3 mutant mice compared to the control mice (Fig. 4d, e).

### Reduction in MIWI2 and MILI loaded piRNAs in the MORC3 mutants

**Figure 5:**
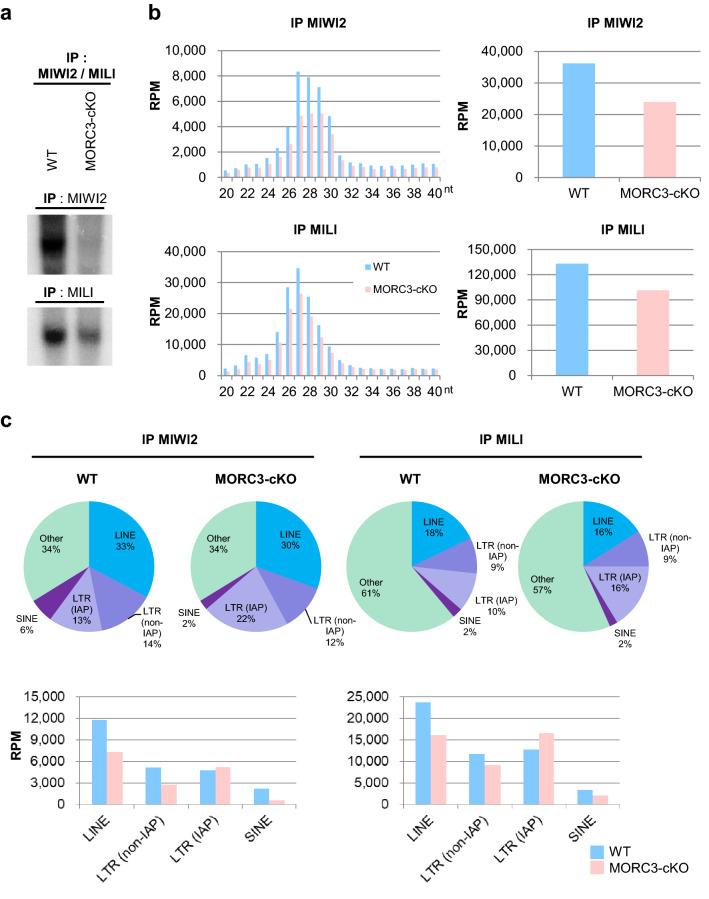

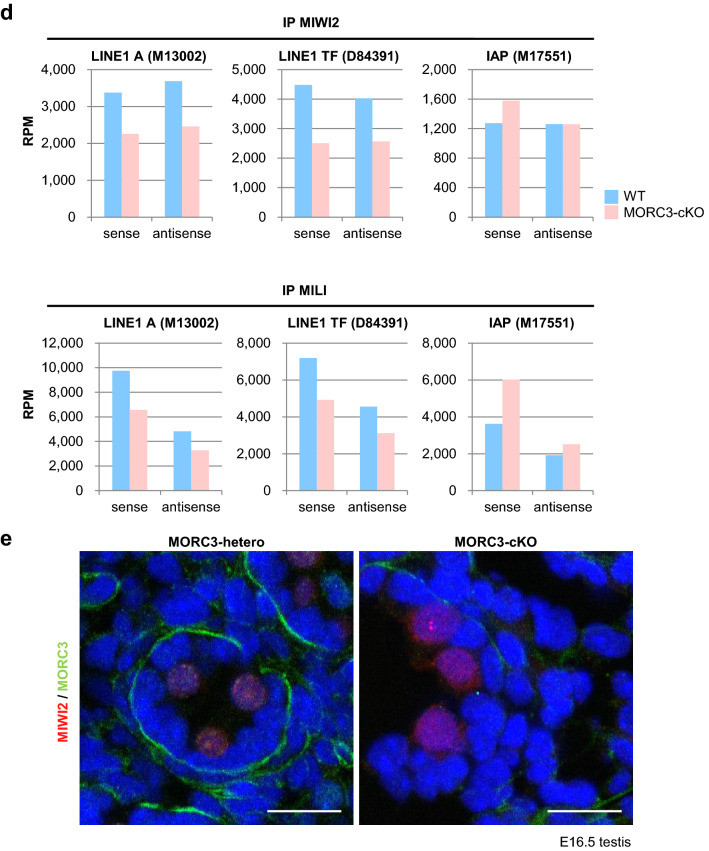
MIWI2- and MILI-associated piRNAs in the MORC3 mutant embryonic testes. (**a**) MIWI2- and MILI-bound piRNAs. The immunoprecipitated RNAs from E16.5 wild type and MORC3-cKO testes were ^32^P-end-labeled and separated in 15% denaturing urea-polyacrylamide gel. Uncropped images were shown in Supplementary Figure S4d. (**b**) Length distribution of small RNAs associated with MIWI2 and MILI from E16.5 wild type and MORC3-cKO testes. The small RNAs were analyzed after ribosomal RNA (rRNA), micro RNA (miRNA), and transfer RNA (tRNA) mapped reads were removed by CLC Genomics Workbench (left). Reads of 25–31 nt small RNAs corresponding to piRNAs in individual samples (right). The accession number of deep sequencing data is DRA009594. (**c**) Genomic annotation and classification of repeat piRNAs based on retrotransposon class in MIWI2- and MILI-associated piRNA libraries from wild type and MORC3-cKO testes (upper). Reads of MIWI2- and MILI-associated piRNAs corresponding to LINE, LTR (non-IAP), LTR (IAP), and SINE in individual repeat piRNAs (bottom). (**d**) Reads of 25–31 nt small RNAs derived from each strand (sense or antisense) corresponding to LINE1 A (M13002), LINE1 TF (D84391), and IAP (M17551) sequences in the MIWI2- and MILI-associated piRNA libraries from wild type and MORC3-cKO testes. (**e**) Co-immunostaining of the testes of the E16.5 MORC3-heterozygous and the mutant mice with anti-MIWI2 antibody (red), anti-MORC3 antibody (green), and DAPI (blue) for DNA are shown. Scale bar, 20 μm.

To analyze the function of MORC3 in more detail, we examined the piRNAs bound to MIWI2 or MILI in embryonic testes. We performed immunoprecipitation of the germ cells of wild type and the MORC3 mutant embryonic testes (E16.5) using the anti-MIWI2 or -MILI antibodies and subjected them to radiolabeled small RNA assay and to deep-sequencing. Western blot analysis revealed that the amount of MIWI2 and MILI proteins immunoprecipitated with the corresponding antibodies were almost the same in the control and the mutant testes (supplementary Fig.S4b and c). However, the levels of MIWI2- and MILI-bound piRNAs were reduced (34% and 24% reduction, respectively) in the MORC3 mutants, compared to the control wild type mice (Fig. 5a, b, and supplementary Fig.S4d-e). Among the MIWI2- or MILI-loaded piRNAs, both the sense and antisense repeat piRNAs mapping to LINE, LTR (non-IAP), and SINE elements were dramatically reduced in the MORC3 mutants. However, the level of piRNAs mapping to IAP (LTR (IAP)) was unaffected in the MORC3 mutants (Fig. 5c, d). The proportion of 1st U sense and 10th A antisense piRNAs was not altered in any category (supplementary Fig.S4f).

Since the entry of MIWI2 into the nucleus is dependent on its association with piRNAs, we examined the localization of MIWI2 in the MORC3 mutant embryonic testes by immunostaining. MIWI2 was still present in the nucleus but the amount of cytoplasmic MIWI2 was higher in the MORC3 mutant testes (E16.5) (Fig. 5e and supplementary Fig.S4g). This phenomenon seems to be corresponding with the reduction of MIWI2-bound piRNAs. In contrast, we observed that the cytoplasmic localization of MILI in the MORC3 mutant embryonic testes was essentially same as that in the control testes (supplementary Fig.S4h).

### Regulation of transcription of embryonic piRNA precursor by MORC3

**Figure 6:**
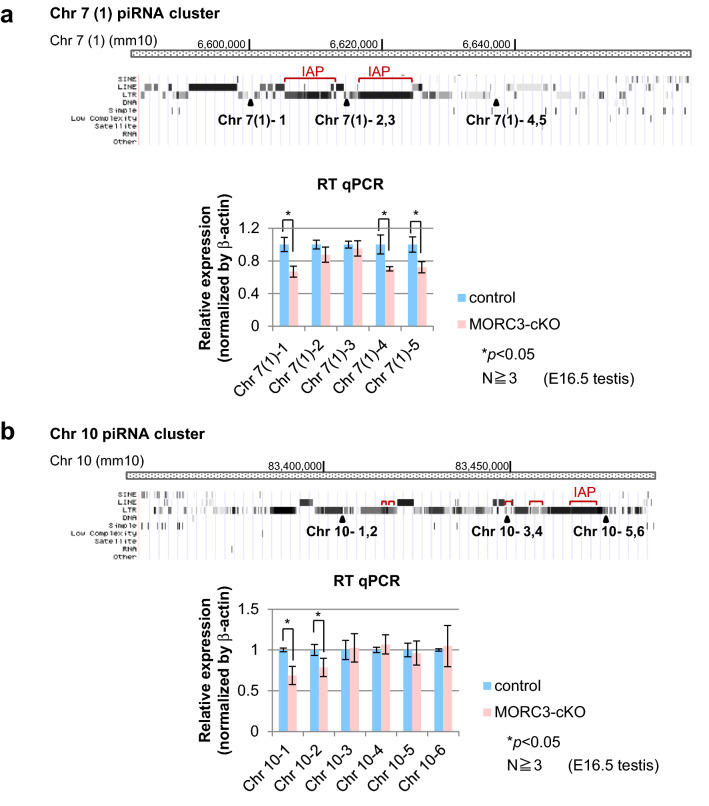

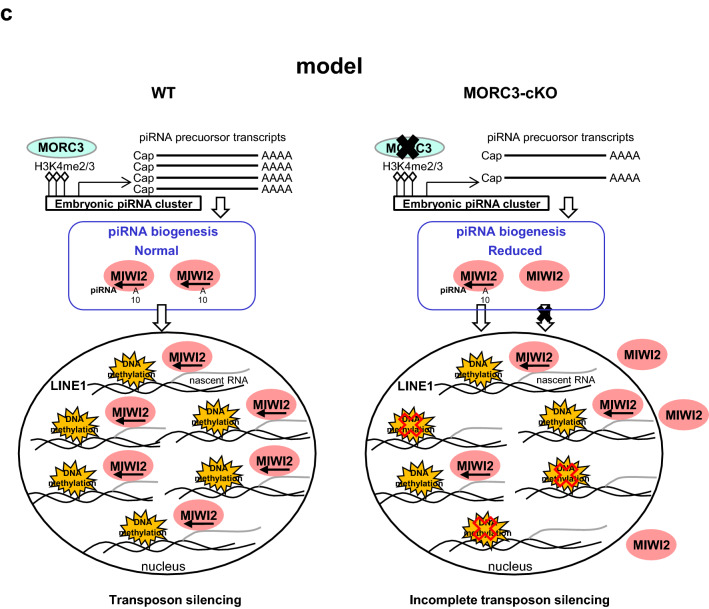
Expression of piRNA precursors from representative embryonic piRNA clusters. (**a** and **b**) Structure of the piRNA clusters (Chr 7 (1) and Chr 10) and positions of individual primer sets. The regions of the IAP element sequence in each cluster are described with red bars (from piRBase in UCSC Genome Browser mm10) (upper). Quantitative RT-PCR for the expression analysis of piRNA precursors transcribed from embryonic piRNA clusters using E16.5 wild type and MORC3-cKO embryonic testes (bottom). Data is normalized by β-actin and is shown as means and SD (Error bar) from more than triplicate PCR reactions. Significant differences (^*^p < 0.05 by the *t* test) between wild type and MORC3-cKO data using primer sets for the Chr 7 (1)-1, 4, and 5 positions (**a**) and Chr 10–1 and 2 positions (**b**) are shown. (**c**) Schematic diagram of de novo DNA methylation pathway mediated piRNAs biogenesis involved with MORC3.

piRNA loci are defined as the piRNA clusters that are regions of the genome mapped with high-density piRNA sequences. We chose some major piRNA clusters in Chr 7, 8, and 10 in the genome, and analyzed piRNA precursor transcript abundance using the wild type and MORC3 mutant embryonic testes by RT-qPCR (quantitative reverse transcription polymerase chain reaction) (Fig. 6a, b). We carefully designed primer sets with unique sequence to identify the transcripts of retrotransposons in piRNA clusters in the genome. For the Chr 7 (1) cluster, Chr 7 (1)-2 and 3 primer sets were located around IAP sequence, and Chr 7 (1)-1, 4, and 5 primer sets were located at a region other than the retrotransposon sequence. Likewise, in Chr 10 cluster, Chr 10-3, 4, 5, and 6 primer sets were located around the retrotransposon sequence whereas Chr 10-1 and 2 primers were not.

The transcript levels of the regions close to LINE, LTR, and SINE sequences were significantly decreased in the MORC3 mutant testes. However, in regions close to IAP the transcript levels were not different between the control and the MORC3 mutant testes. Essentially, similar patterns were also found in Chr 7 (2) and 8 clusters (supplementary Fig.S5a and b). These data show that MORC3 has some retrotransposon class-specific effect to regulate the transcription of piRNA precursor from embryonic piRNA clusters.

## Discussion

Several reports have shown the importance of the role played by MORC family proteins in retrotransposon expression. Homologues of *Morc* are required for transposon silencing in *Arabidopsis* and *C. elegans*. Furthermore, *Morc1*, a *Morc* mouse homologue, is the epigenetic regulator of piRNA-independent transposon gene silencing^35,36,40,41^. Results of the current study identified MORC3 as a component of the MIWI2 protein complex and a regulator of transposon repression in male germ cells. MORC3 is essential for repression of retrotransposon via de novo DNA methylation involved in the piRNA pathway. Herein, we show that MORC3 is a new player in piRNA biogenesis and acts as an epigenetic regulator of transposon gene silencing via the piRNA pathway.

Immunoprecipitation of the sample from the ZF-MIWI2 Tg testis with anti-Flag antibody and subsequent mass spectrometry analysis revealed a physical association between ZF-MIWI2 and MORC3 (Fig. 1a and supplementary Fig.S1a). This association likely involves direct binding, as the experiments using 293T cells expressing MORC3 and MIWI2 confirmed the binding phenomena (Fig. 1d and supplementary Fig.S1d-i). Basically, the other MIWI2 binding components expressed in male embryonic germ cells are scarcely expressed under this condition. We found that MORC3 expression in the fetal testis was restricted to germ cells and the protein was predominantly localized to the nucleus and piP-body. This localization pattern is quite similar to that of MIWI2 (Fig. 1e and supplementary Fig.S1l).

The data of physical binding of MORC3 with MIWI2 prompted us to determine the physiological role of MORC3. We noticed that the DNA methylation level of some classes of retrotransposons was significantly lower and both MIWI2- and MILI-associating piRNAs were reduced by about half in the MORC3 mutant embryonic testes (Figs. 3, 4, 5). Thus, MORC3 is at least partially required for piRNA-directed de novo DNA methylation. It is conceivable that the loss of MIWI2 loaded piRNAs would be the reason for the incomplete de novo DNA methylation in the MORC3 mutants (Fig. 6c).

MORC3 mutants lead to de-silencing of LINE1 but there was no-effect on IAP expression. The other proteins involved in piRNA production, such as TDRD1, TDRD9, TDRKH, and EXD1, also regulate LINE1 expression rather than IAP^17–19,45,46^. In contrast, in MILI and MVH mutants, de-suppression of IAP was observed to be as robust as that of LINE1^12,15,20^. The loss of piRNA-dependent DNA methylation, which was detected in MILI- and MVH-null testes, leads to meiotic arrest at the pachytene stage in spermatogenesis^20,47^. In contrast, the vast majority of the MORC3 mutant male germ cells escape such apoptosis and survive through the meiotic prophase (Fig. 2a, b). However, the pregnancy rate of the normal female mice crossed with the MORC3 mutant male mice was significantly lower and the fertility of the MORC3 mutant male mice was slightly impaired (Fig. 2c and supplementary Fig.S2g).

Comparing to the phenotype of the MORC3 mutant mice, impaired fertility was not observed in EXD1 mutant mice, despite the reduction of MIWI2-bound piRNAs, especially against LINE1. The reason for the normal fertility in EXD1 mutant mice could be the milder effect of de-repression of retrotransposons^46^. Taken together, the high survival rate of the MORC3 mutant germ cells in spermatogenesis is presumably due to the milder degree of the deregulation of LINE1 and reduced levels of piRNAs, compared to those in MILI- and MVH- null mutants. Although it is difficult to conclude that the fertility defect of the MORC3 mutant male mice is dependent on the impaired retrotransposon expression, some association may be anticipated.

Lastly, we wanted to understand the mechanism by which the impaired retrotransposon expression was effected in the MORC3 mutant testis. Previous studies revealed that piRNA precursors are conventional RNA pol II transcripts bearing 5′ caps and 3′ poly (A) tails. The lengths of these nascent RNAs, which are transcribed from transcriptional start sites in the piRNA clusters, are varied^27^. The coordinated increase of pachytene piRNA precursor transcripts is controlled by common transcriptional factors, such as the A-MYB protein, during spermatogenesis, especially around the pachytene stage. It has been suggested that this regulation would be caused by the compartmentalization and reorganization of TAD (topologically associating domain)^27,48^. However, the mechanism of transcriptional regulation for embryonic piRNA precursors remains unclear.

Our RT-qPCR data indicated that transcription of piRNA precursors originated from retrotransposon regions in piRNA clusters and that the transcripts except those of the IAP regions were significantly reduced in the MORC3 mutant embryonic testis (Fig. 6a, b, and supplementary Fig.S5a and b). These data support the notion that the regulatory mechanism of transcription including IAP regions on the piRNA clusters is different from that of transcription from the other retrotransposon regions in gonocytes.

The H3K4me3 (H3 tri-methylated lysine 4) signals, that are histone modification associated with RNA pol II transcription start sites, are highly observed in promoters of retrotransposons including piRNA clusters in E16 testes^49^. In addition, the promoter regions of piRNA clusters show much stronger H3K4me3 signals than coding gene promoters in embryonic testis^50^. MORC3 has the PHD X/ZF CW domain, which recognizes and binds to H3K4me3 marks, and the GHKL ATPase domain, which is involved in ATP binding and hydrolysis^51,52^. Enrichment of MORC3 is found in H3K4me3 sites of active gene promoters in the genome-wide ES cell analysis^52^. We confirmed that the accumulation of MORC3 was observed in the promoter region of piRNA cluster (supplementary Fig.S5c). Taken these observations together, our findings suggested that MORC3 might recognize and bind preferentially to H3K4me3 marks at the promoter region of not only retrotransposon genes but also piRNA clusters and regulate the transcription of piRNA precursors via chromatin remodeling by hydrolyzing ATP in embryonic testis (Fig. 6c).

Considering that MIWI2 is localized at the promoter regions of retrotransposons, it is speculated that MORC3 proteins would be localized to the similar regions through a protein complex formation and would play a role in the embryonic transposon silencing via piRNA pathway^32^. Compared to wild type testes, the reduction of MIWI2-bound piRNAs was only slightly larger than that of MILI-bound piRNAs in the MORC3 mutant embryonic testes Meanwhile, there were no difference in the degree of 10th A bias in total antisense piRNAs between the mutant and wild type testes, suggesting that MORC3 might be also partially involved in the transfer of secondary piRNAs to MIWI2 in the piP-body (Figs. 4e, 5a–d, and supplementary Fig.S4b-f). Therefore, notably, MORC3 would have a major contribution to the initial stages of piRNA biogenesis rather than subsequent secondary piRNA pathway, in which MIWI2 is involved. Thus, MORC3 affects not only secondary but also primary piRNA biogenesis and regulates the transcription of piRNA precursors.

MIWI2 affects the chromatin state by targeting nascent RNAs transcribed from piRNA-dependent retrotransposon regions using piRNAs as guides. The piRNAs may be generated in part from the nascent RNAs transcribed by the regulation of MORC3. Thus, MIWI2 plays the role as a recruiter of proteins related to de novo DNA methylation on retrotransposons, while MORC3 controls the transcription of piRNA precursors from retrotransposon related piRNA cluster regions of the entire genome. MORC3 and MIWI2 might mostly play a separate role on piRNA-dependent retrotransposon regions, at the first step of primary piRNA biogenesis and after secondary piRNA biogenesis, respectively. Considering the report from the other group that MIWI2 might have some involvement in the primary piRNA biogenesis, it is conceivable that MIWI2 might have some effect on the transcriptional regulation of piRNA precursors with MORC3, which is chromatin remodeling factor, at least in part^53^.

In this study, we have identified MORC3 as a nuclear effector of retrotransposon silencing via piRNA-dependent de novo DNA methylation. Our data suggest that MORC3 acts as an active regulator of piRNA precursors in the piRNA pathway and subsequently affects piRNA production. Thus, we provide the first mechanistic insights into effector protein at the first step of piRNA biogenesis in embryonic transposon silencing mechanism.

## Methods

HE staining, immunoprecipitation, SDS-PAGE, and silver staining, isolation of sperm, Northern blot analysis, Immunohistochemical staining, Detection of small RNAs, ChIP-qPCR assay, Sperm morphology and motility, and primers are described in the Supplementary Information.

These study protocols were approved by the Gene Modification Experiments Safety Committee and the Animal Experiment Committee of the Osaka University medical school.

### Animals

All the animal experiments were performed in accordance with the general guidelines of The Institute of Experimental Animal Science, Osaka University Medical School. This study is reported in accordance with ARRIVE guidelines. The transgenic mice carrying the *FLAG-NLS-ZFP* and *FLAG-NLS-ZFP-MIWI2* transgene were previously reported^32^. *Morc3* conditional knock out mice were generated using *Morc3* floxed mice (gift from Dr Haruhiko Koseki (RIKEN Center for Integrative Medical Sciences) and Tnap-Cre transgenic mice (gift from Dr Andras Nagy^43^). Founder mice were mated with C57Bl/6 mice to generate the lines. The transgenic mice and *Morc3* conditional knock out mice were validated by PCR with specific primers.

### Generation of *Morc3* conditional knockout mice

We generated conditional knockout mouse lines in which we inserted the targeting vector (pDTMorc3neo) from the bacterial artificial chromosome clone (RP23-119J20) containing mouse genomic DNA using the double Red recombination method as described previously^54^. This targeting vector was constructed to produce an appropriate conditional allele for Morc3*.* Parallel LoxP sites were inserted such that they flanked a critical portion of the gene: the LoxP sites flanked exon 2 (containing a part of Histidine kinase/HSP90-like ATPase superfamily domain) of Morc3 (supplementary Fig.S6). The DNA sequence of the targeting vector (pDTMorc3neo) is shown in supplementary item S1. The linearized targeting vector was introduced into mouse ES cells (lab-made ES cells originated from hybrid embryos derived from C57BL/6J and C57BL/6N mice) by electroporation (GenePulser; Bio-Rad). The ES cells were grown on feeder cells in an appropriate medium. Each colony was picked up and expanded, and the genomic DNA of each clone was purified. To identify true homologous recombinant colonies, we performed direct sequencing through PCR amplification using the outward primers for the homologous region and the inward primers for the neomycin resistance gene. Targeted ES cells were used for generating chimeric mice. The resulting chimeric mice were backcrossed to C57BL/6J mice for over 5 generations.

### Isolation of testicular germ cells

Whole testes (D12 after birth) were removed and treated with 1 mg/mL collagenase type II and DNase1 for 15 min at 37 °C. These samples were suspended with 0.25% trypsin for 10 min at 37 °C and washed with Hank’s Stock Solutions (HBSS) (Nacalai Tesque Inc., Kyoto, Japan). Anti-EpCAM (PE) antibody (CD326) (G8.8, BioLegend) was added to the cell suspension with 5% BSA/PBS. After stirring for 90 min at 4 °C, the immune complex was washed with HBSS. The germ cells were sorted using a BD FACSAria system (BD Biosciences, Franklin Lakes, NJ, USA) after immunostaining with the anti-EpCAM antibody. The germ cell purity was verified by rerunning the sample after sorting and was determined to be > 90%.

### Bisulfite sequencing

Sorted germ cells (D12 after birth) were treated with bisulfite using the EpiTect Fast DNA Bisulfite Kit (Qiagen, Valencia, CA, USA). The primers that we used for the bisulfite sequencing of type A LINE-1 and type TF LINE-1 loci in this experiment recognize more than several thousand loci in the mouse genome. Meanwhile, among IAP retrotransposon species, only 1Δ1-type IAP, which is only 5–6% of total IAP elements, shows piRNA-dependent DNA methylation in 5′-LTR^55^. Unfortunately, 5′-LTR sequences of 1Δ1-type IAP could not be distinguished from those of the other types of IAP. Therefore, we have used the primers specifically recognizing one locus of 1Δ1-type IAP (in this experiment, a locus in Chromosome 3). The PCR primer sequences are described below. The first and second rounds of PCR amplification of the IAP and the *H19* (GenBank accession no. U19619) were carried out using Ex Taq (Takara, Japan). The PCR conditions were as follows: an initial round of 2 min at 94 °C followed by 30 cycles of 30 s at 94 °C, 30 s at 50 °C (for H19) or 1 min at 55 °C (for IAP), and 1 min at 68 °C, and the second round of 2 min at 95 °C followed by 15 cycles of 30 s at 95 °C, 30 s at 50 °C (for H19) or 1 min at 56 °C (for IAP), and 1 min at 72 °C. Nested PCR was performed to amplify the *H19* differentially methylated regions (DMRs). The PCR for the type A LINE-1 (GenBank accession. no. M13002) and type TF LINE-1 (GenBank acc. no. D84391) was carried out using EpiTaq HS (for bisulfite-treated DNA) (Takara, Japan) under the following conditions: 2 min at 94 °C followed by 30 cycles of 30 s at 94 °C, 30 s at 55 °C (for type A LINE-1) or 30 s at 50 °C (for type TF LINE-1), and 1 min at 68 °C. The PCR products were purified using the QIAquick Gel Extraction Kit (Qiagen, Valencia, CA, USA), cloned into the pGEM-T Easy Vector (Promega, Madison, WI, USA), and sequenced using an Applied Biosystems 3730 DNA Analyzer (Thermo Fisher Scientific, CA, USA).

### Western blotting

The immunoprecipitates or lysates were separated by SDS-PAGE (7.5% gel) and transferred to a PVDF membrane (Millipore, Bedford, MA, USA). After blocking, the filters were incubated with an anti-MIWI2 monoclonal antibody (25D11), anti-MIWI2 polyclonal antibody (MIWI2-C), anti-MILI antibody (26F)^47^, anti-MILI antibody (PM044; MBL, Japan), anti-MORC3 antibody (D238-3; MBL, Japan), anti-MORC3 antibody (100–401-N97; Rockland), anti-PA antibody (Wako, Japan), anti-FLAG M2 antibody (Sigma Chemical Co., St. Louis, MO, USA), anti-FLAG antibody (FLA1; MBL, Japan), or β-actin antibody (Sigma 5441). Anti-MIWI2 monoclonal antibody (25D11) was produced with GKGRQDFEELGVC (a 69–80 aa peptide sequence of MIWI2) as the antigen by Dr Yasuyuki Kurihara (Yokohara National University). An anti-MIWI2-C polyclonal antibody was generated by immunization with peptides derived from MIWI2 (amino acids 831–847: SVHKEPSLELANNLFYL). HRP anti-mouse IgG and HRP anti-rabbit IgG (Pierce, Rockford, IL, USA) were used as the secondary antibody, and the signal was detected using ECL Western Blotting detection reagents (GE Healthcare, Chalfont, Bucks, UK).

### Mass spectrometry

Mass spectrometry was performed with the UltiMate 3000 Nano LC system, Q-Exactive (Thermo Fisher Scientific) and analyzed with MASCOT software (Mascot Distiller v2.5, Mascot Server v2.5, www.matrixscience.com) (Matrix Science, London, UK) by CoMIT Omics Center in Osaka University.

### RT-qPCR

Total RNAs prepared from E16.5 testes were treated with Turbo DNase (Life Technologies) and subjected to RT-PCR using SuperScript III Reverse Transcriptase (Invitrogen) and Oligo (dT), and to Q-PCR using THUNDERBIRD SYBR qPCR MIX (TOYOBO, Osaka, Japan). The qPCR was analyzed using the CFX384 Real-Time PCR system (BIO-RAD), and the specific primers used are described in the Supplementary information.

### piRNA preparation and deep sequence analysis

Total RNA samples were prepared from E16.5 testes of the *Morc3* homozygous mutant and wild type (control) mice using ISOGEN (Nippon Gene CO., LTD., Tokyo, Japan). Small RNA Library was generated with TruSeq small RNA Library Prep Kit and analyzed with Illumina HiSeq (Macrogen Inc). For immunoprecipitation of MILI or MIWI2 piRNA complex, 38 testes from the *Morc3* homozygous mutant and wild type (control) mice at E16.5 were collected, respectively. The collected testes were homogenized in lysis buffer (20 mM HEPES (pH 7.3), 150 mM NaCl, 2.5 mM MgCl_2_, 0.1% NP40, 1 mM DTT) containing protease inhibitor tablet (Roche) and RNasin (Promega). The piRNA complexes were immunoprecipitated using 14 testes by anti-MILI (PM044, MBL) and using 24 testes by anti-MIWI2 (Miwi2-N1)^47^ antibodies, respectively. Then, the samples were subjected to RNA purification using ISOGEN-LS (Nippon Gene CO., LTD., Tokyo, Japan) and to western blotting using anti-MILI (26F) and anti-MIWI2 (25D11) antibodies. The small RNA Libraries were generated with TruSeq small RNA Library Prep Kit and analyzed with Illumina NovaSeq. This work was supported by Japan Society for the Promotion of Science (JSPS). The raw sequence data in all samples were processed by CLC Genomics Workbench software (version 12, https://digitalinsights.qiagen.com/products-overview/discovery-insights-portfolio/qiagen-clc-genomics/) (Qiagen) to trim of miRNA (miRbase), rRNA (5S rRNA Database), and tRNA (GtRNAdb) allowing up to 2 mismatches after removing adaptor sequence, low-quality reads, and reads below length (15 nt) and above length (45nt). The trimmed 25–31 nt length small RNA reads (corresponding to the length of piRNAs) were mapped to repetitive DNA consensus sequence using Dfam database allowing up to 3 mismatches after mapping the mouse genome allowing 0 mismatches (UCSC/mm10) and mapped to L1A sequence (M13002), L1TF sequence (D84391), and IAP sequence (M17551) allowing up to 2 mismatches. The number of mapped read counts was normalized by the read-depth of each library.

### Plasmid, Cell culturing and transient transfection

The 293T cells were cultured in Dulbecco’s modified Eagle’s medium (DMEM) supplemented with 10% FCS. The 293T cells were transfected with PA-tagged MIWI2 plasmid^49^, Myc-His-tagged MILI plasmid^56^, and Flag-tagged MORC3 plasmid (gift from Dr Norimitsu Inoue (Department of Tumor Immunology Molecular Genetics, Osaka Medical Center for Cancer and Cardiovascular Disease, Osaka, Japan)^2^, using polyethylenimine (Cosmo Bio, Tokyo, Japan) according to the manufacturer’s instructions. At 27 h post-transfection, the cells were harvested by centrifugation for 10 min at 3000 × rpm at 4 °C.

### Male fertility test

Sexually mature mutant male mice were caged with 7-week-old wild type (C57Bl/6) females for several months. A check for the vaginal plug was carried out every morning, and the number of pups in the cage was counted.

## Supplementary Information

Supplementary Information.
