# Supplementary material for: MORC3, a novel MIWI2 association partner, as an epigenetic regulator of piRNA dependent transposon silencing in male germ cells
Source: Sci Rep. 2021 Oct 14;11:20472. doi: 10.1038/s41598-021-98940-7 (PMC8516955; doi:10.1038/s41598-021-98940-7)
Supplement: Supplementary file 1 — Supplementary Information. [file 41598_2021_98940_MOESM1_ESM.pdf]

Supplementary Information
Supplementary Figure S1

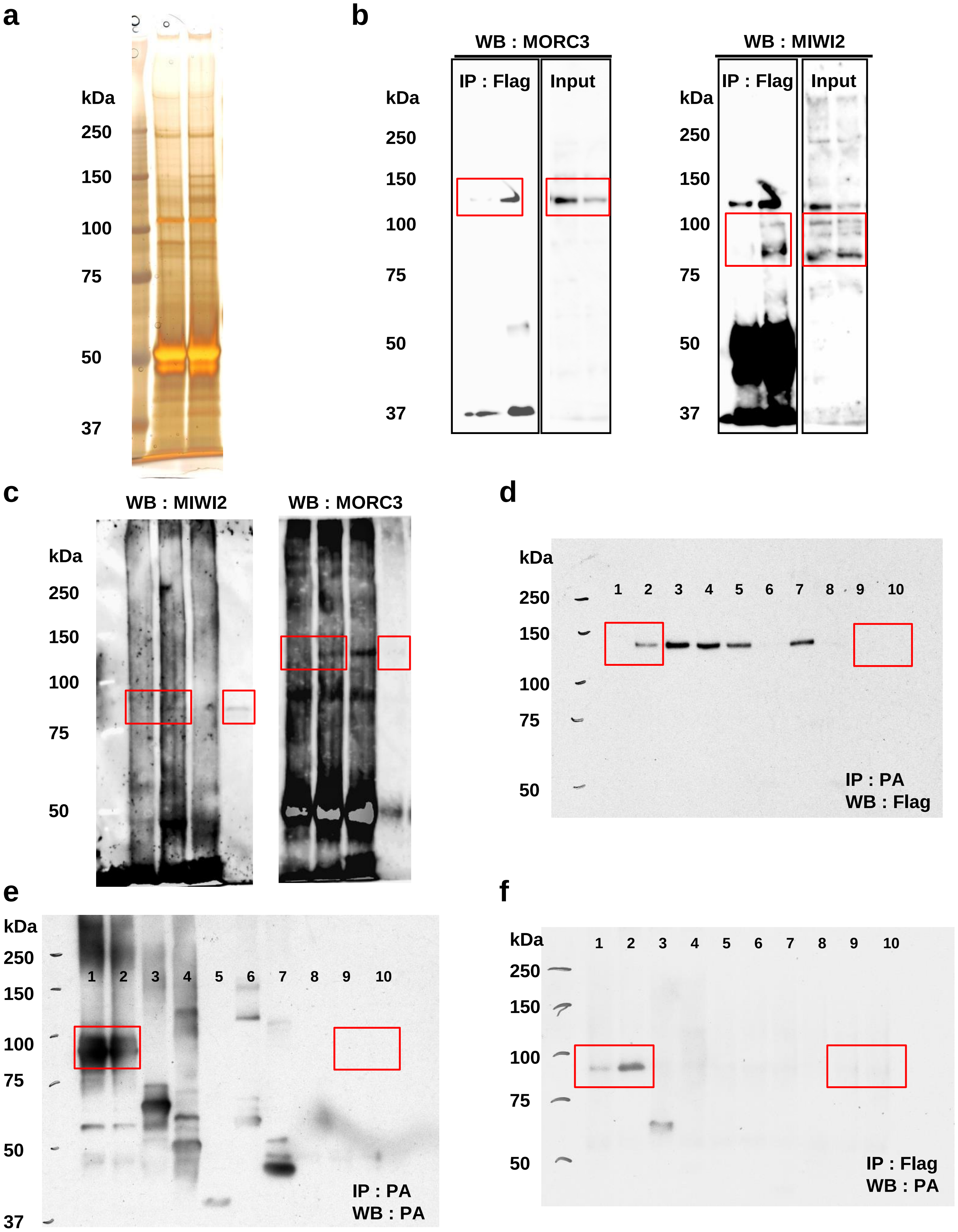

Supplementary Figure S1

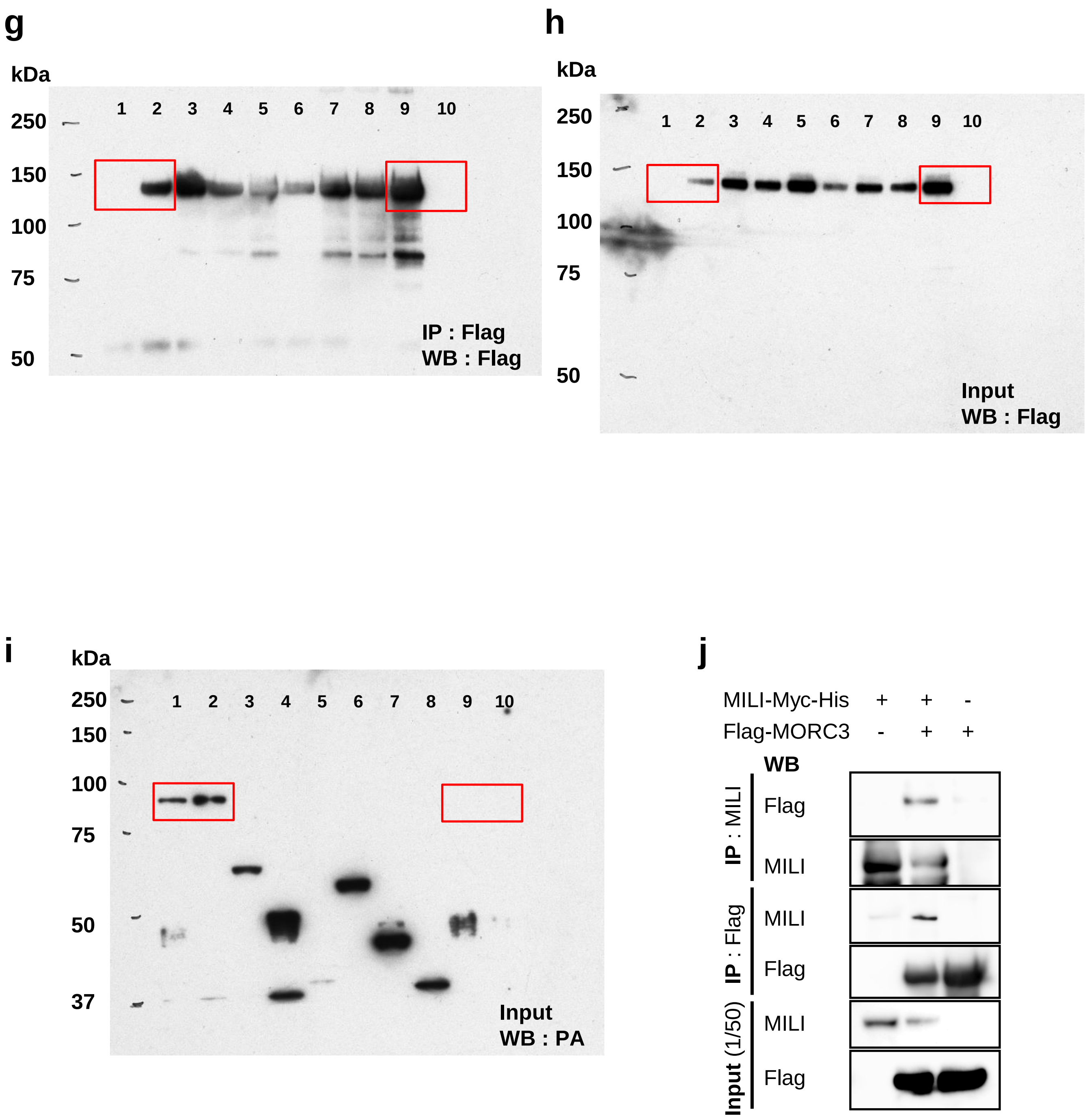

Supplementary Figure S1

k

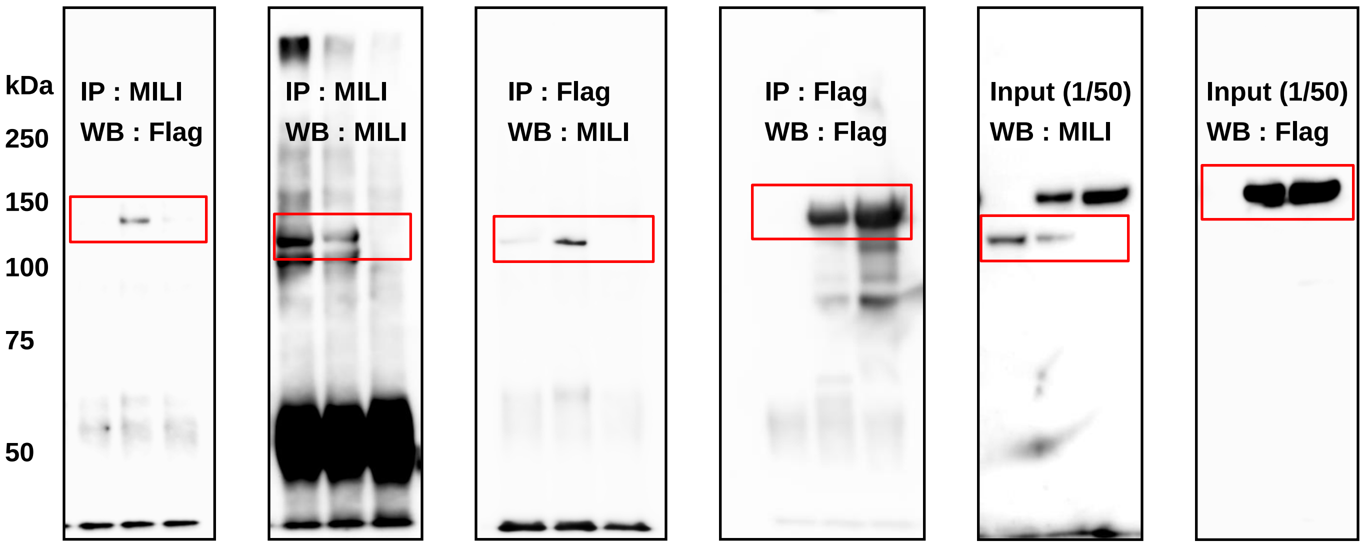

l

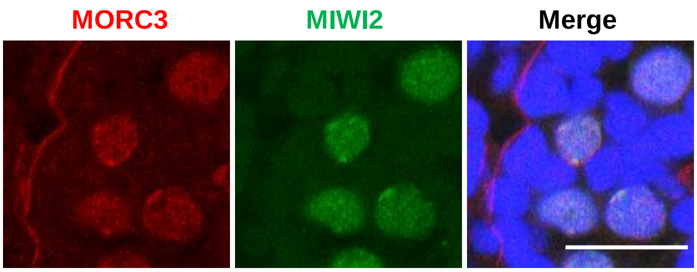

Supplementary Figure S2

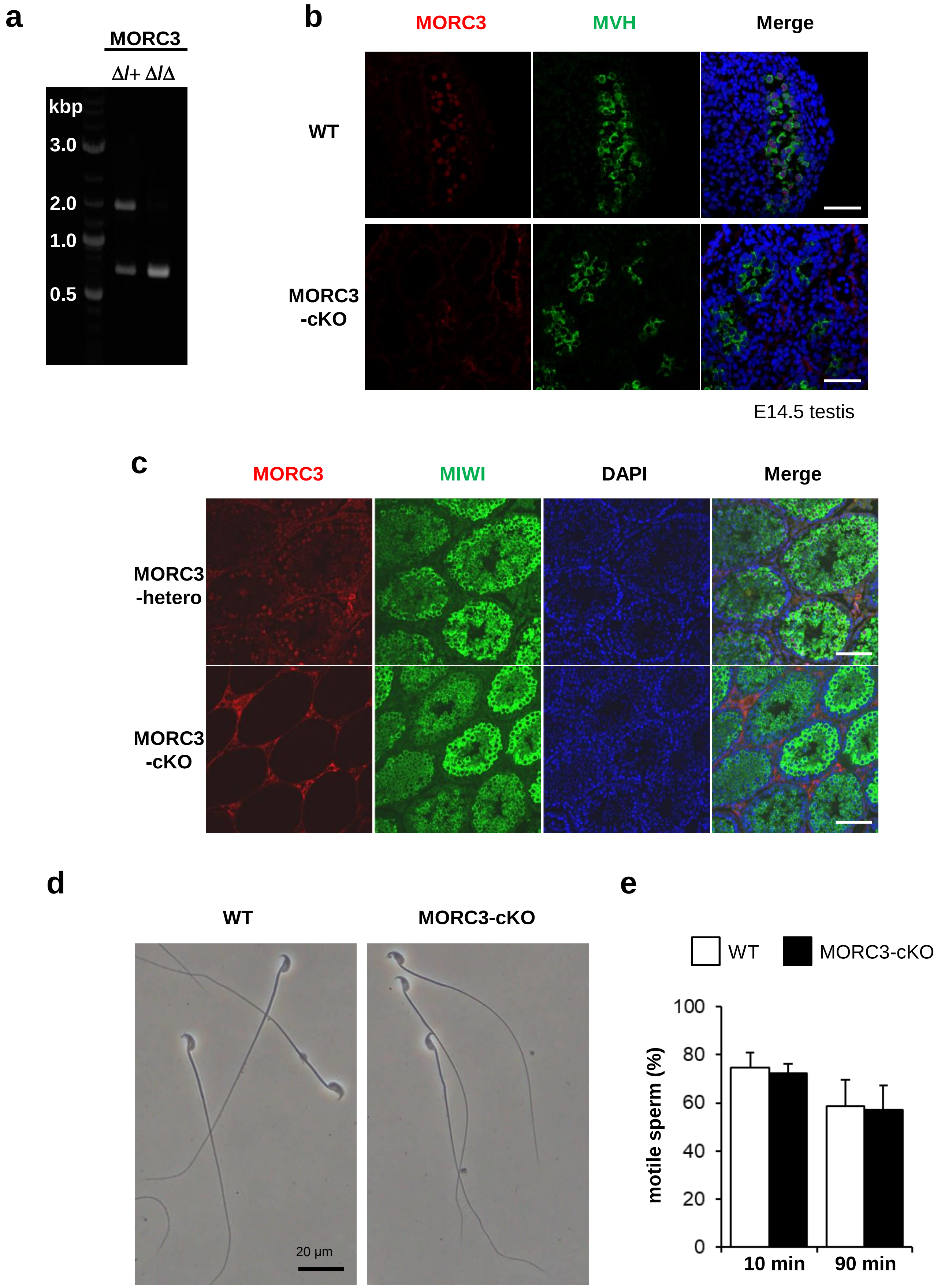

Supplementary Figure S2

f

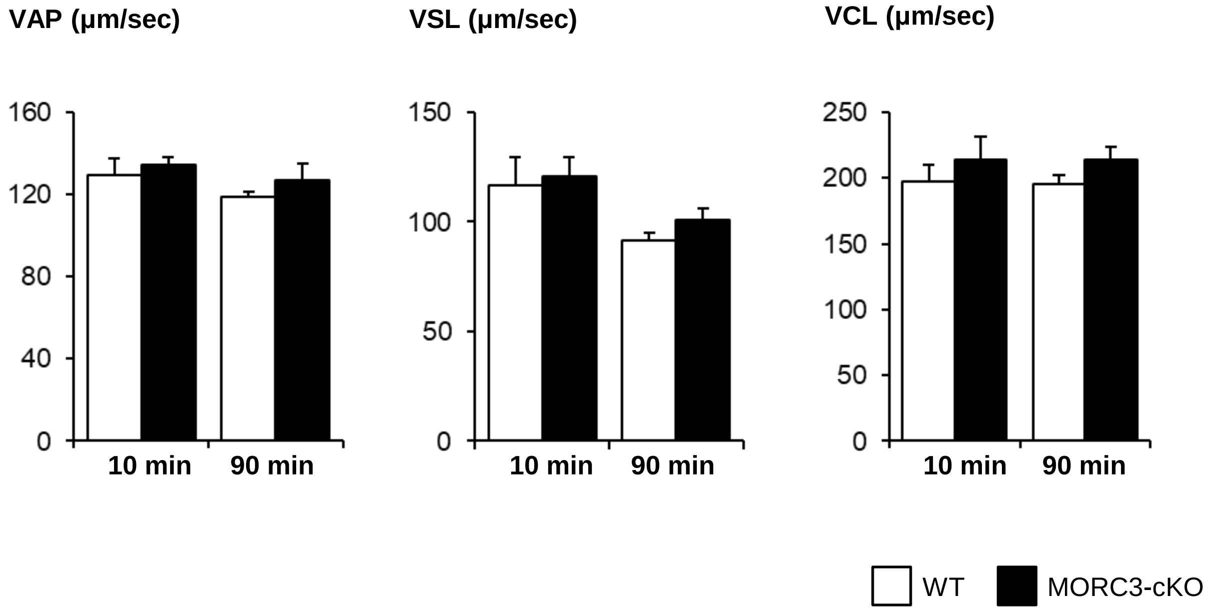

g

	♂No.	plug (No.)	birth (No.)	litter size
MORC3-cKO	#116	31	2	10,7
	#118	26	9	7,7,7,5,5,6,1,6,4
	#120	26	7	8,9,9,5,10,4,6
	#122	10	1	6
	#125	31	0	
	#127	5	4	9,8,7,6
	#198	15	4	6,8,8,7
	#203	17	0	
	#208	16	5	7,7,6,6,6
	#232	16	8	6,6,9,4,5,8,7,7
	#238	27	0	
WT	#1	8	8	7,9,9,6,6,10,9,8
	#2	5	4	8,9,7,5
	#3	5	4	7,7,6,9

Supplementary Figure S3

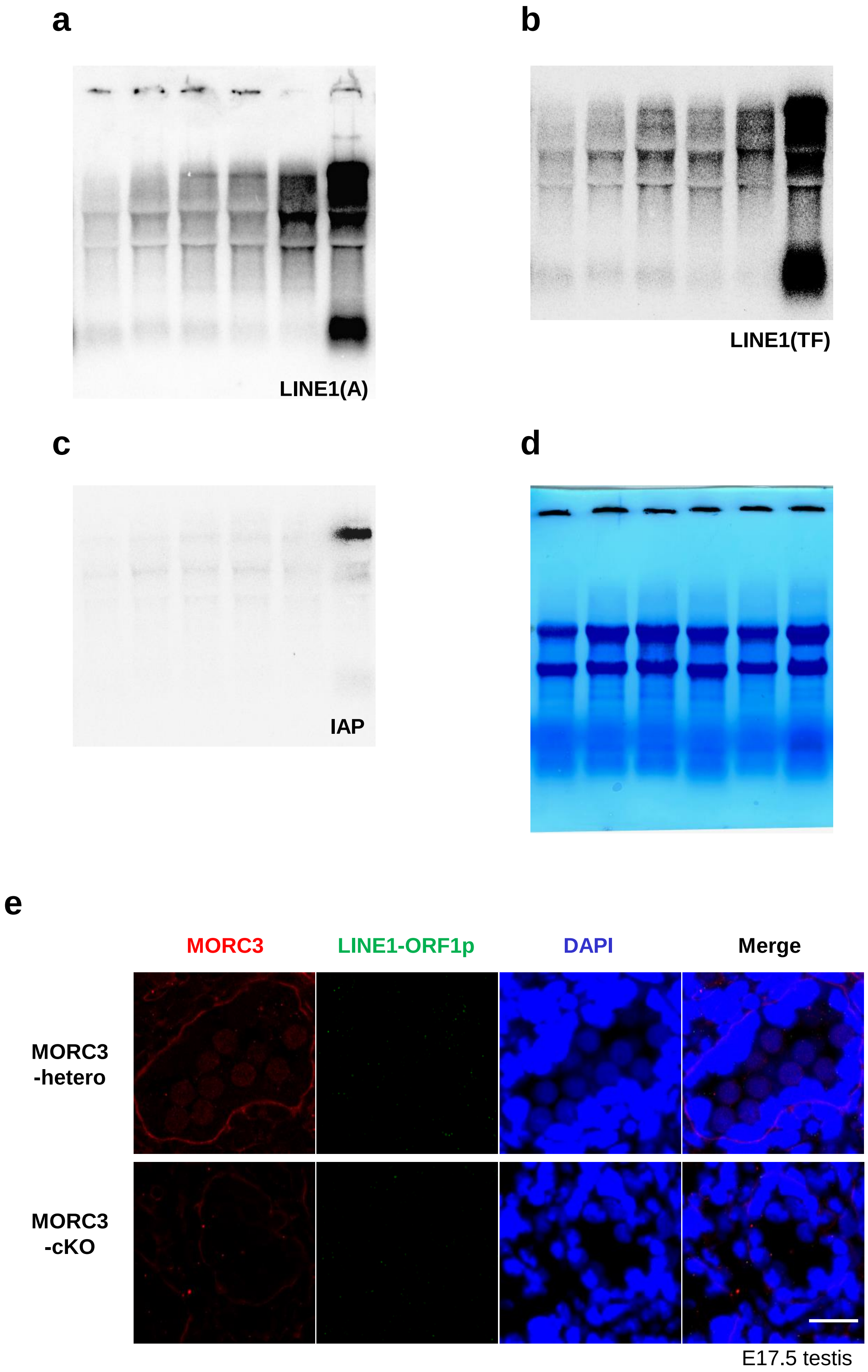

Supplementary Figure S4

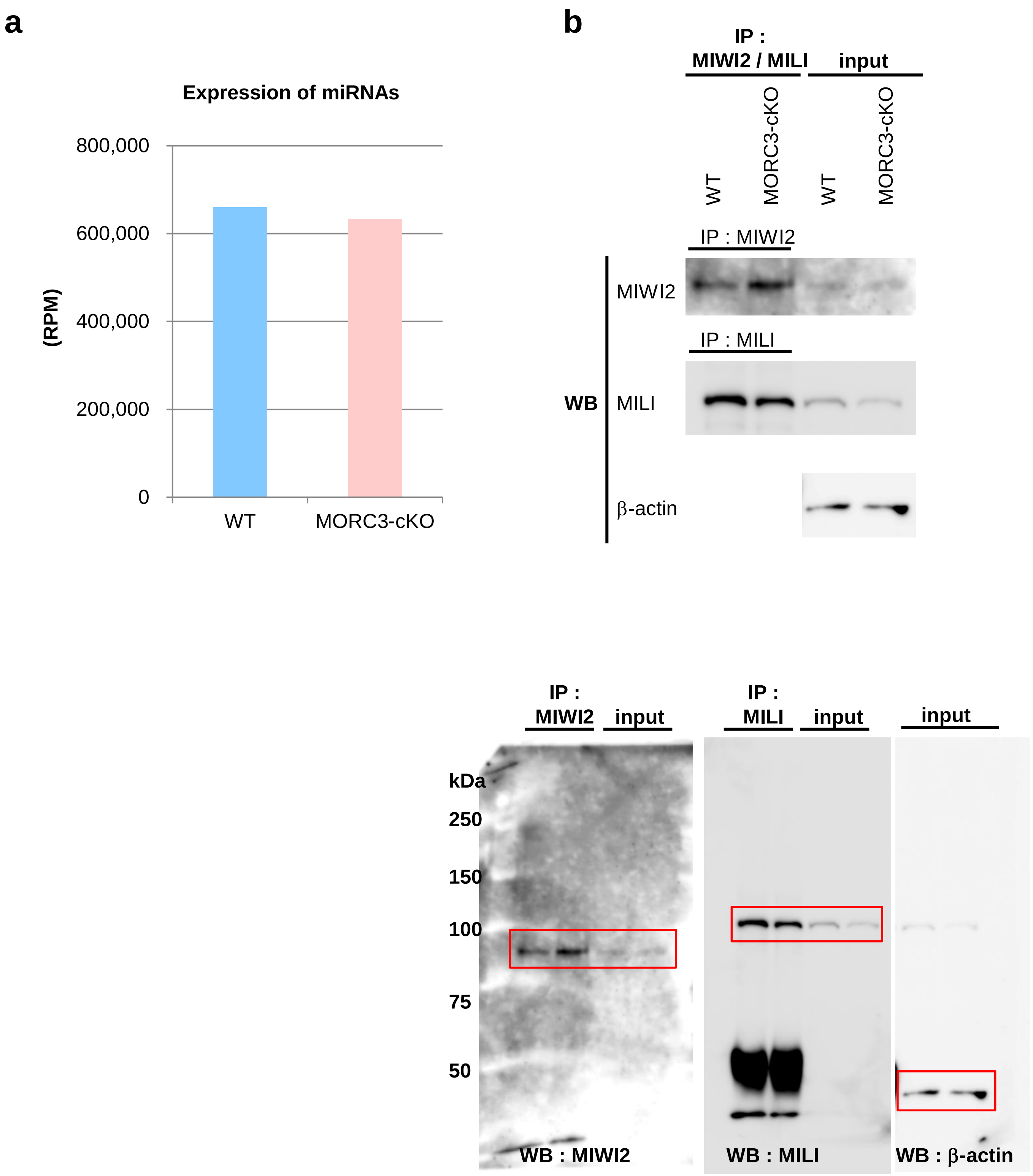

Supplementary Figure S4

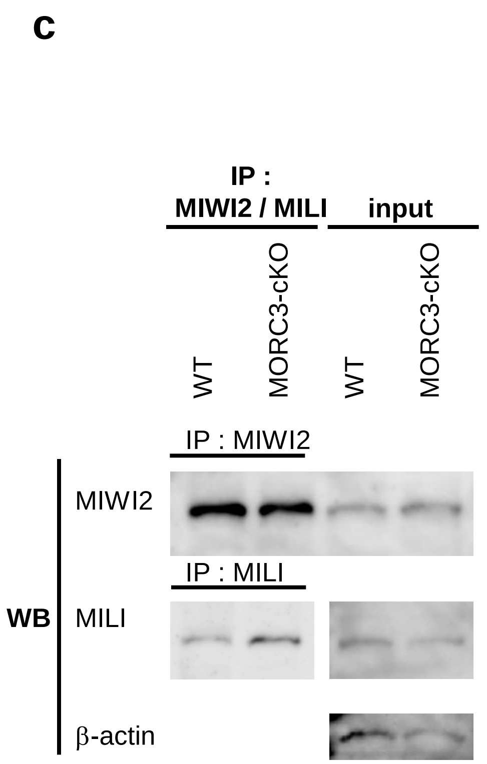

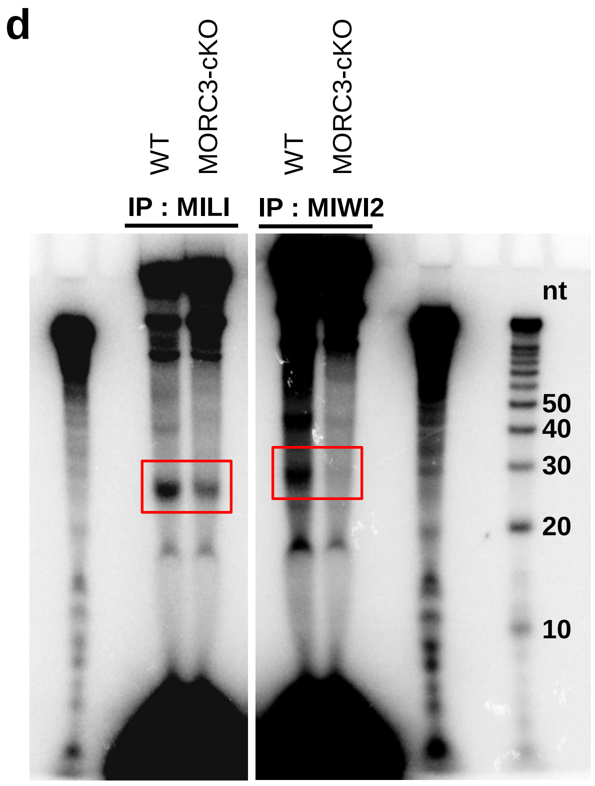

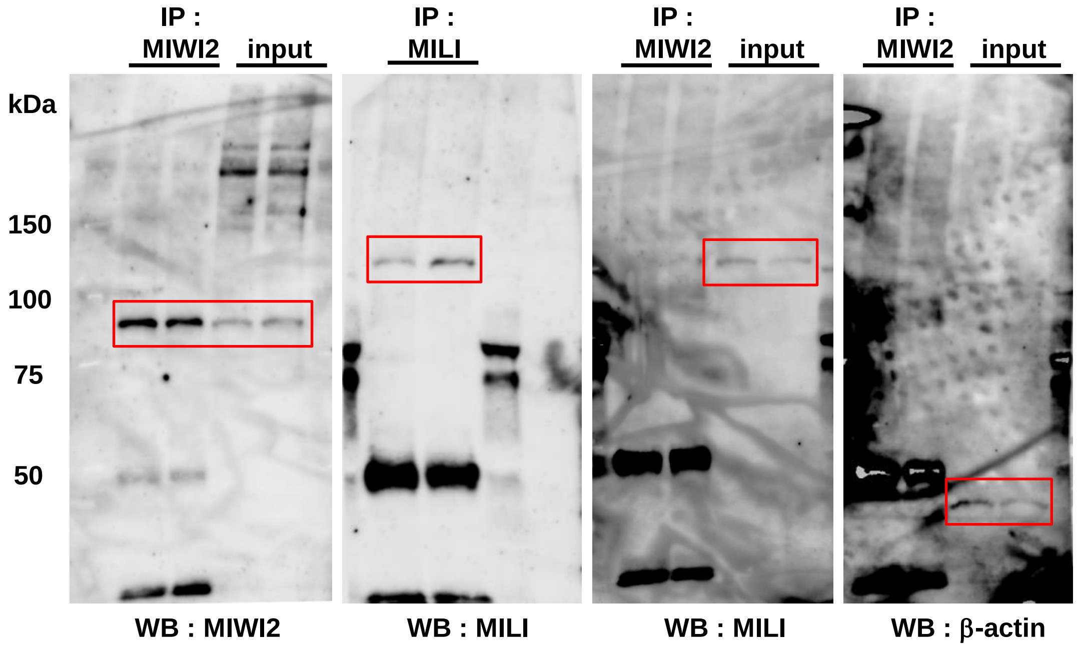

Supplementary Figure S4

e

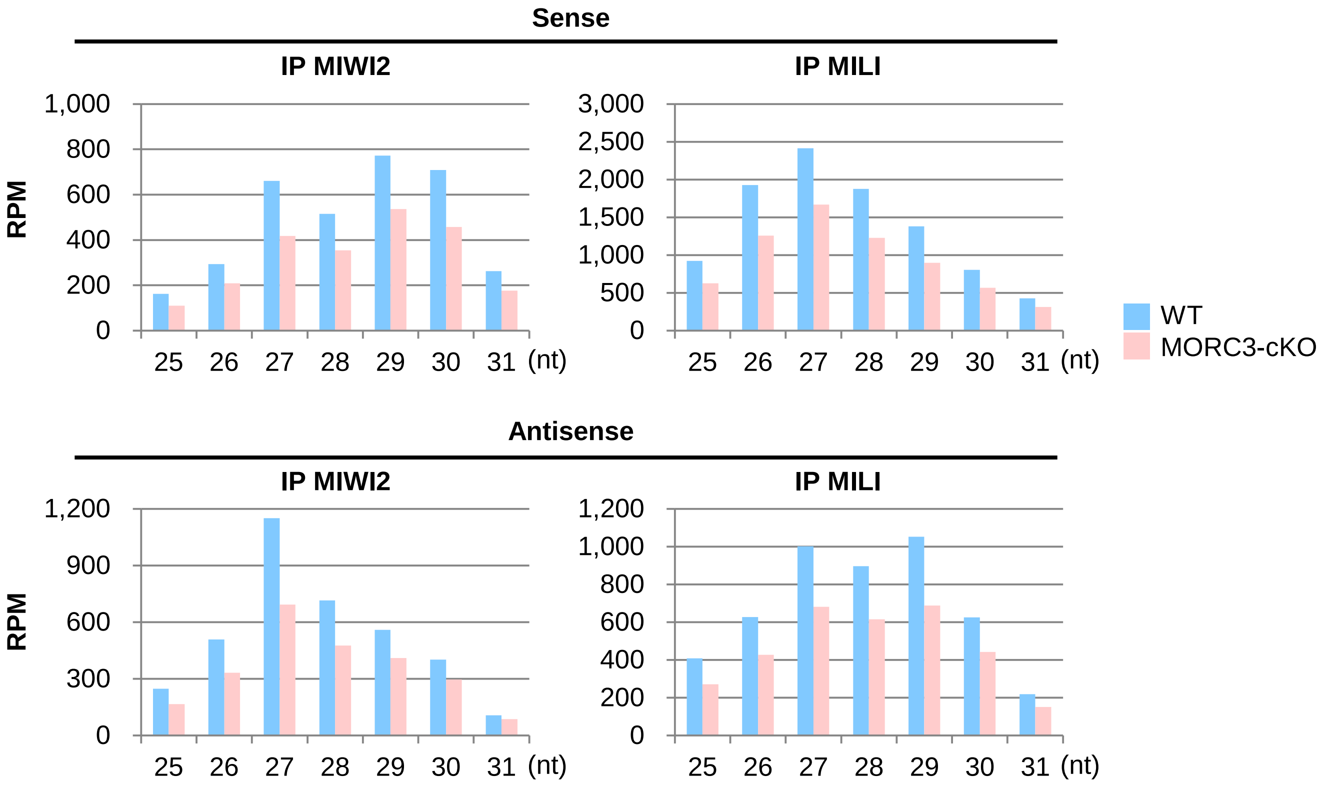

f

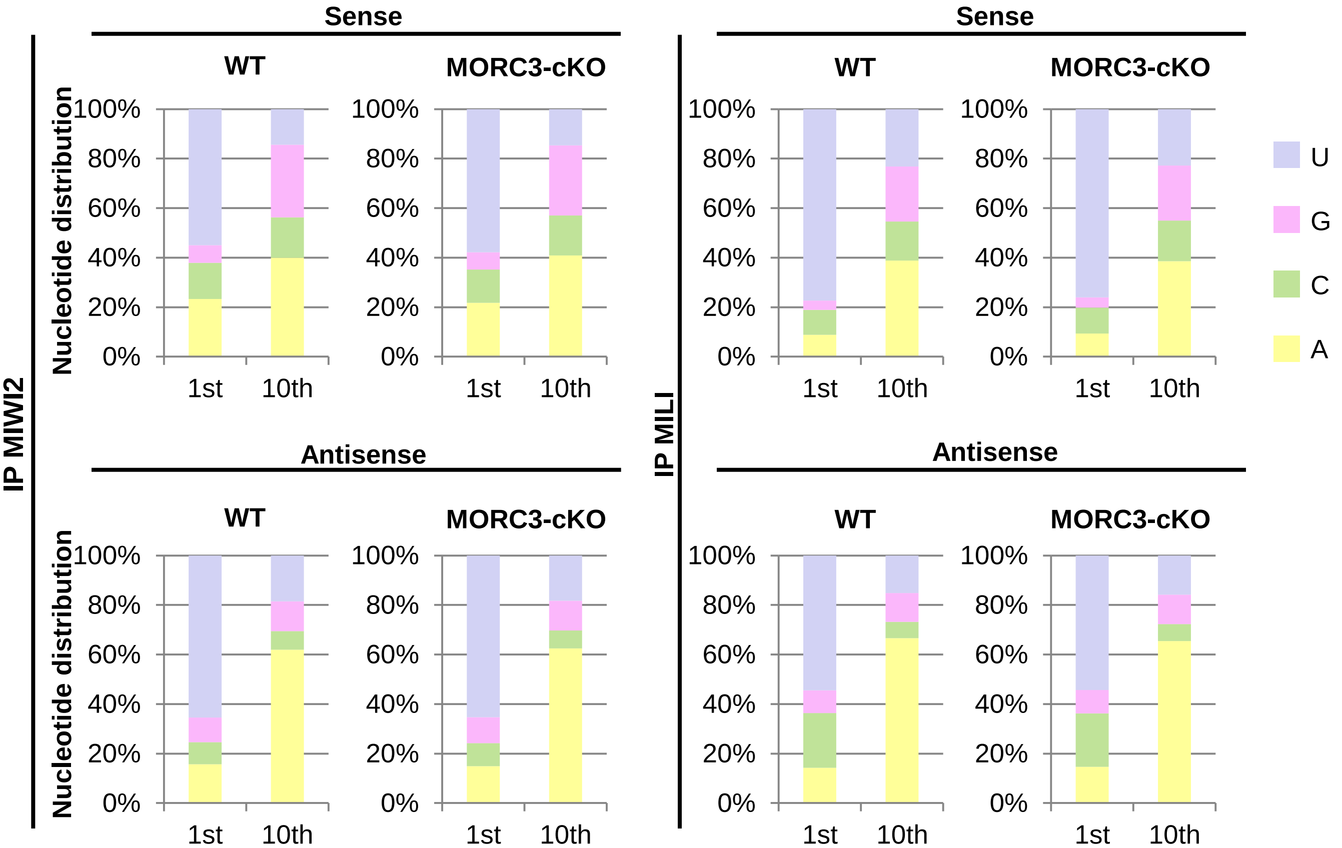

Supplementary Figure S4

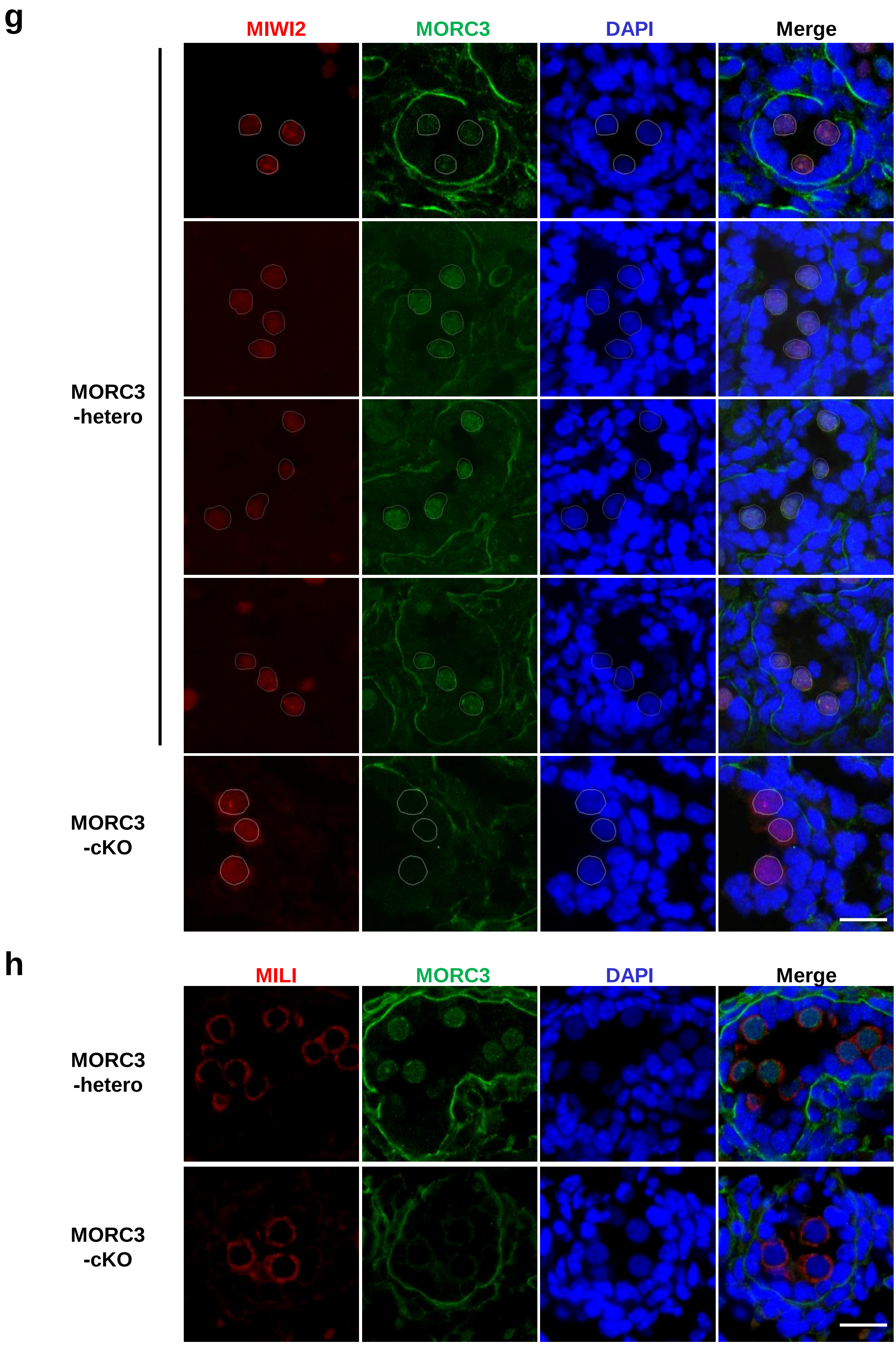

Supplementary Figure S5

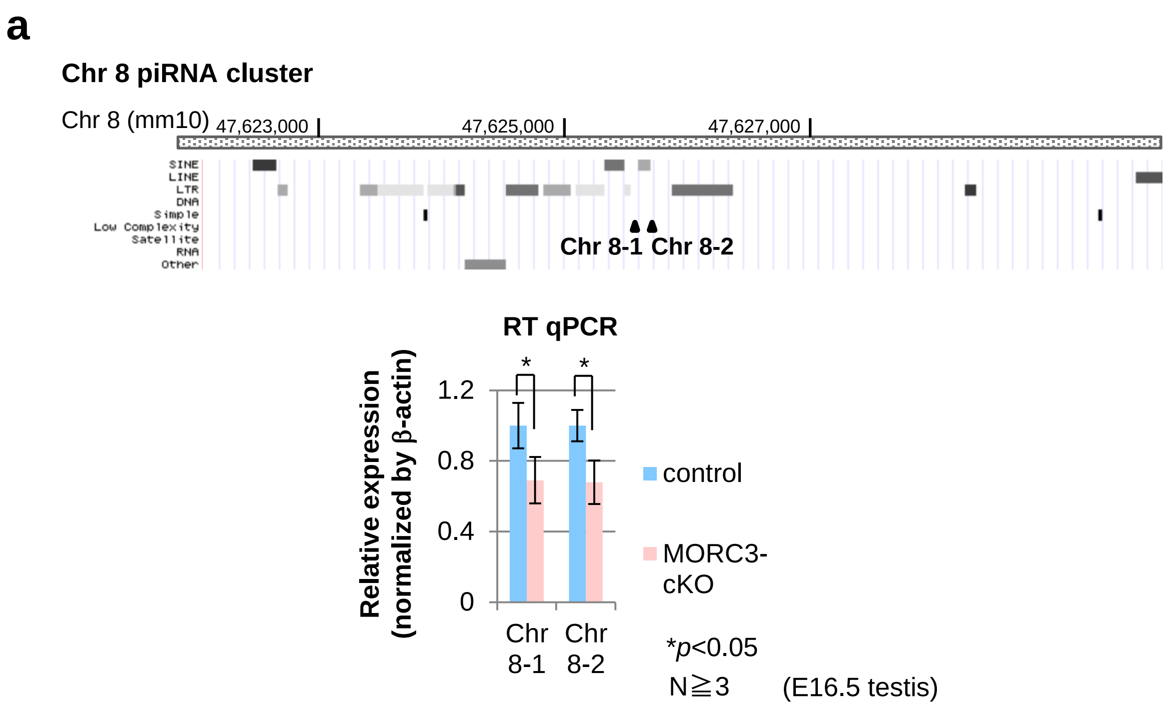

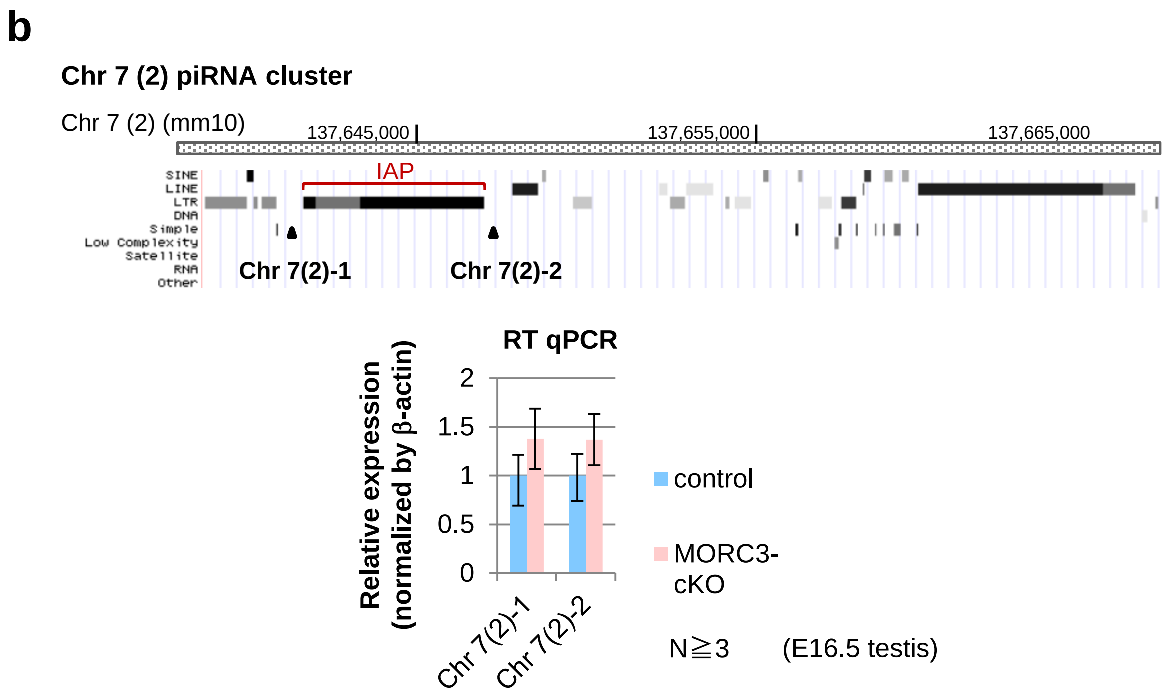

Supplementary Figure S5

c

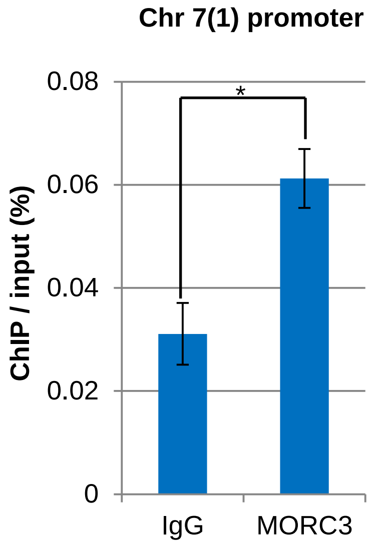

*$p<0.01$
N=3
(E16.5 testis)

Supplementary Figure S6

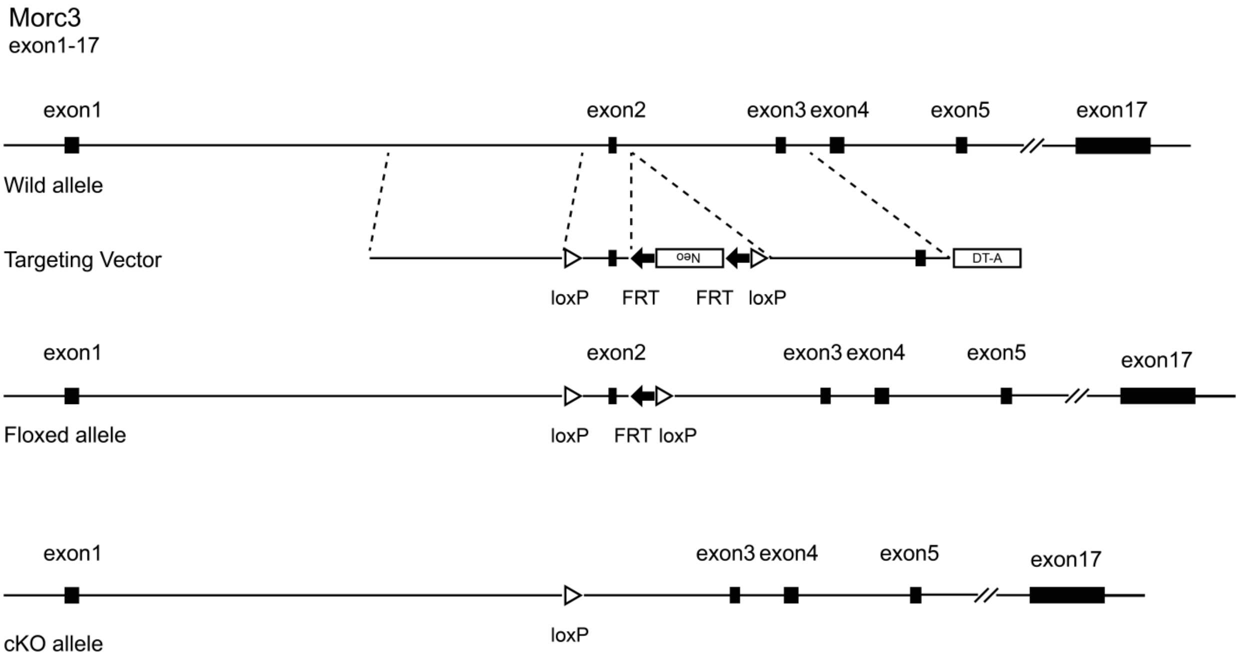

Supplementary Information

Supplementary Item S1

> pDTMorc3neo

CCACCTAAATTGTAAGCGGCCGCTACAATAGACCAAGATGGTCAGACTGACAGAGA
TTCTCTACCTTCCAGGAGCTGGAATAAAAGCCATGTACGCCTAGCTAGCCTCCTGC
AACTGTTTAGAGTTGGTGGGGTTTTGTTGTTGTTGTTGTTGTTGTTTTGTTTGT
TTTTGTTTTTTTGAGAGAGAGCATGTGAGAGAGCGTGAGCTAGAGTTTGCGTGTGG
AAGCCAGGAAAGGCAATTGCTTTGCTTAGAGCTGGAGCTACAGTCACTTAGGGGCT
TCTCGAGGAATTTCAATTCAGAACATGGCTGCTATGGCAAGAAATCACATCCAAAA
AAAAAAATCTTCTAGGTCTGTGTTATCTGGCTGGAATACAGAGGAACCTTGAATCC
AAGGAGACAGGCAAGCAGATCTCTGAGTTCAAGGTCAGCCTTGTATAGAGCAACAT
TCGGTAAAGAAAAGCTTAGGTCCAGGCATGGAGGCGCACACCTTTAACCCAGTACT
CAGGTGACAGAGGCAGGTGGATGTCTGAGTTCTGTTCAGTCAGTTCATGAAAATCA
GAGGCAGCTATCAGTAGAGTTATACAGAGAGAGGTTGAAGAGAGAGCAAGCTAGAT
ACAAATAAAGACCGAACAAGCCAGAGAATTAGAAGGAGCCAAAAGATTAGAACAAA
TTGCTAGAGTTACTTTGAGACCAAGCAGGGCAATTCAGTGAGAAGCAGAAAGAAG
CCAGTTTTGAGTCATCTTGAAGAGGAGTTTTGAGCCAGAACAGCTGAATTAATGCA
GTCAGCCAGAGCTCAGGAAGAACTAGGAAGGGTGAGCTTACTCAGTGGTAAGTCT
CAGGCTGAGCATTCTCAGCCTAGGTTAGCAGACAGAGGCAGTAAGCCCCAGACAG
CAATTGTATCAGGAGAATAAAAAGTATAGAAACTGACCAGTGTGACCCTGAGAACC
AAACTCTGGTTTAAGCTTTATTGAATTCTGAAAGTTGAATTTTTTTTTTTTAAGTTTT
TGCTTTTCTCTGGTGCTAAGATGAAACCCAAGATGCTGTAACTGCTGGACAAGTAC
CCTGCCACCAGTGTCTGTCCCCAGGGTTTTCACTGTCATTTGTTTTGTGATTGAGG
TTTCCTGGGAAAAGCTGGGACTTCGAAGGCCAATTGACATCAGTGATGTGGATTCA
GAGTTCAGAGCCAAGTTACCTTAAGACATCTTTGGCCCTTTAATAGCCATTATTGCC
CTCCCCTGTGGCTTAAGGTCTTTGAGCATTGGGAAAATCATTTGAAGTATATGGATG
GACCCCTATATTTCTCTCAACCAAAAGGTCAAGGAATGCTGTCCAAAGCATCTGAG
GCTGAAAACAGCATTTTCCTAGCTTTACTGTAACCTACCAGCTAGTGTACCCAAAAG
CCATGATACTTGGATTCCTTTGCTCTGCTACTCTAAAAATTTCATCAGATTATTACTA
CTCTTGAACATTTGGCTCTAGAATGAACTGTCTCTTACCCCTTTGGGGTGAGAGCT
GTTTCTTACGTTGCTGGTGAACTTAGATGTACAGTCATAAATGTCACTTTCATTTCT
TCAGCTGAAGTGATGAGATAAGAGCTGTACCCAAGCTGTGCAAATACATGGCTTGG

GCATTTCCTTCTTTGTTCTAACTATCCCTAAGTCTGGTCCTAGAAGCTTCCCACCTC
TGTACAATATAATCTTCCAAGGTGAATGATTCATTCTGGCTGCTCTTGGCTTCTGAC
TGAATTGTTCTGCCTGGCCTCATACTAACTTTGACAATCTGTCCTAATCTCCTTCTT
GTTCTCTGGCTTGTCCTCTCTGTAACCTATCTCTGTAGAACTGTCTCTGTGAAACTG
CCACTCACCACACATACCACACACACATACACACACACATCACGGGCACACACACT
GCACACCTTTTTCTCACTCTCTCTGCTCTTTATGTAGTCTCTCTCCCTCTGCTGTTC
TCATGTGAACTGAGAGTATCCTATCTCTGACAACTCTTTCTCTGATTGGTCACTTTG
CCACTCAATTAGATGCCACTTTCAAACATTGGCTGCTTCCTTCTACAAACAAACCTT
ACCTTCACTGTTAGGGATTAAAGATGTGTACTAAGGGTGTGTCTGTATTCTGGCTG
GATCATATCTTTGGATGTGATCTCTTGCTAGAGCAGCCATGTTGCTGGATTAAAATT
CCTCTACATACATAGTTCTAAAATTTGATATAGTTTGATTTATATGCCTTTTTTTGTTA
TATTCAAGAAATAATTCCCAAATCAAATGTCTTGAAGCTTTTTTCTTCTAAAGTCTAA
CAATTTATCTCTTATTCTAGGTGGGTTTTGTTGTTATTGATGGTGATGGCAGTGTTT
TGTTTTGTTTTTTTTTTCCGAGACCAGATTTCTTTGTGTACCTCTGGCTGTCCTGGA
ACTCACTCTGTAGACCAGGCTGGCCTCGAACTCAGAAATCTGCCTGCCTCTGCCTC
CTGAGTGCTGGGATTAAAGGTGTGCGCCACCATGCTGGGCTATTTTAGGTTTTGAT
CTATTTTGACGTAGTTTCTATGTATTGTAATGCAGGAATTTGGTTTGCCCAGTTCCA
TTTGTTGAAAGGGCTTTTTCCAGTGAAGATTTTGGTGCCATATGTTCAGGGTCTGT
TTGTAGGCTGCTGACTTGTTGTGAGTCTGCCTGTCTTAGCGGTCCTTGGAGTCAGC
TTGCCATGTCCATTCCTGTAGGGAGCTGATCTCTGCAGTTGTCTGGGTTTTCTGTA
GCCACATCTCCAACAGCTCATGCTGGGTGCCAACTTAAAACTTGCTTATGTTTTGAT
ACTCTGCTTTCCTAGTGATGGAATTATAGGCTCTTGGTCACCATGCTCAGTTGTACG
GAGTGCTGGGAGTTGAACCCAGGGCTTTGTGAATGTTAGGCAGGCGTTCCACCAA
TTGAGCTACATCCCCAACCTTTTGTGTTGGGGAAGTGGAGGCAAGAGGATTCGAA
GTTTAAGGGGAGTTCAAGGCTATCCTAGGATACGTGACCCTGTCTTTTTTTTTTTTT
TTAAGTGTTTAAACTAACTTATTACGTGTGTATGCCACCAAATGTTCAAGAAATTAA
GTTTCCTATTGGCTTCTCCTTCCATACCCTATTATTGAGAGTAAAGATGTACTTTAAA
ACCTTGGAAAACTTAAAAACCTTCATTTGTTGTTGGAAGTAATATTTGAGATTCTTT
GAAGAGGCCATGGATGTACACAGGCATAACTTCGTATAGCATACATTATACGAAGTT
ATCACTGTTTGACTGTAAGAAACTTGGAATAGAAAATAAATCCAACTTGAGGTGTC
AGATATTTAGTCTCTGAAAGGCAACCAGCAAAGCCAGTAGATGAAGCAAAGCTAGT
AAGGTCATTACACTGATGACCAAATACCTTCTTGGCAAGAGTTTGTCTCAAAGGAA
GGATTTAGGAACCGGTGTTGGTGGGTCCTTAGGGTCTCGGTTGGGTCATTTAAAAC

AAGTCTTACAAAGGAATTGGGAATTAGGCAAGCTCTGCATTGTGGACACAAGGAGA
CAGGGTTTTAAGAAGGGCTCCATGTGAACATTATTGAGTTATTTCTCCAGCCTTATC
TTCCAGGAAGCCTCTAGGTTTTATCTTCTTGGAGCAAGCAATCAGACATTTTTTTAT
TTGGCATCAATGTTGTTTAACACAGTTTTTATTGGGTCTTGTGCTTATAATTAAGGG
CTGAATTAAACTTTATGAATTGTTGGTTTGTTTCAGCTTTGCCCGAAGTTTTTACAT
ACAAATTCTACTAGTCATACCTGGCCGTTCAGTGCAGTTGCTGAGTTAATAGGTATG
TACATTGTTGTTTCATTATGTCTCTTAATTGTAATTTTAGTTGACTTCAGAAAAATAA
TATGCAATTATGCCTTTTCAAAGTGTATCCTGAGTAACAGTGTGCATTCCTACTTAG
ACGTCGTTTAAGTTGCTTTCAGGTGGATTACTCCTCAGCCTTTCCTGTATTACTCAA
GTGCTTCATTGCTTTTAGCACTTAATTTGCTAAGTTGACATAGCACTTACGGTGAAG
TTCCTATACTTTCTAGAGAATAGGAACTTCCGCGCCGCACACAAAAACCAACACAC
AGATCATGAAAATAAAGCTCTTTTATTGGTACCGAATTCGCCAGGGAGCTCTCAGA
CGTCGCTTGGTCGGTCTTTATTCGAACCCCAGAGTCCCGCTCAGAAGAACTCGTCA
AGAAGGCGATAGAAGGCGATGCGCTGCGAATCGGGAGCGGCGATACCGTAAAGCA
CGAGGAAGCGGTCAGCCCATTCGCCGCCAAGCTCTTCAGCAATATCACGGGTAGC
CAACGCTATGTCCTGATAGCGGTCCGCCACACCCAGCCGGCCACAGTCGATGAATC
CAGAAAAGCGGCCATTTTCCACCATGATATTCGGCAAGCAGGCATCGCCATGGGTC
ACGACGAGATCCTCGCCGTCGGGCATGCGCGCCTTGAGCCTGGCGAACAGTTCGG
CTGGCGCGAGCCCCTGATGCTCTTCGTCCAGATCATCCTGATCGACAAGACCGGCT
TCCATCCGAGTACGTGCTCGCTCGATGCGATGTTTCGCTTGGTGGTCGAATGGGCA
GGTAGCCGGATCAAGCGTATGCAGCCGCCGCATTGCATCAGCCATGATGGATACTT
TCTCGGCAGGAGCAAGGTGAGATGACAGGAGATCCTGCCCCGGCACTTCGCCCAA
TAGCAGCCAGTCCCTTCCCGCTTCAGTGACAACGTCGAGCACAGCTGCGCAAGGA
ACGCCCGTCGTGGCCAGCCACGATAGCCGCGCTGCCTCGTCCTGCAGTTCATTCA
GGGCACCGGACAGGTCGGTCTTGACAAAAAGAACCGGGCGCCCCTGCGCTGACAG
CCGGAACACGGCGGCATCAGAGCAGCCGATCGTCTGTTGTGCCCAGTCATAGCCG
AATAGCCTCTCCACCCAAGCGGCCGGAGAACCTGCGTGCAATCCATCTTGTTCAAT
GGCCGATCCCATGGTTTAGTTCCTCACCTTGTCGTATTATACTATGCCGATATACTAT
GCCGATGATTAATTGTCAACACGTGCTGCTGCAGGTCGAAAGGCCCGGAGATGAG
GAAGAGGAGAACAGCGCGGCAGACGTGCGCTTTTGAAGCGTGCAGAATGCCGGG
CCTCCGGAGGACCTTCGGGCGCCCGCCCCGCCCCTGAGCCCGCCCCTGAGCCCGC
CCCCGGACCCACCCCTTCCCAGCCTCTGAGCCCAGAAAGCGAAGGAGCAAAGCTG
CTATTGGCCGCTGCCCCAAAGGCCTACCCGCTTCCATTGCTCAGCGGTGCTGTCCA

TCTGCACGAGACTAGTGAGACGTGCTACTTCCATTTGTCACGTCCTGCACGACGCG
AGCTGCGGGGCGGGGGGGAACTTCCTGACTAGGGGAGGAGTGGAAGGTGGCGCG
AAGGGGCCACCAAAGAACGGAGCCGGTTGGCGCCTACCGGTGGATGTGGAATGTG
TGCGAGGCCAGAGGCCACTTGTGTAGCGCCAAGTGCCCAGCGGGGCTGCTAAAGC
GCATGCTCCAGACTGCCTTGGGAAAAGCGCCTCCCCTACCCGGTAGAATGAAGTTC
CTATACTTTCTAGAGAATAGGAACTTCAAGCTTATAACTTCGTATAGCATACATTATA
CGAAGTTATGAAAATATTTATATTCTTATGAAAGTAAAAAAATAAGAATTACACTTTC
CAGTACTGTGTGTACCATCCAGAGAGGTTTCATCTTTCAGGTGGTATTGCATTTATG
ACAGTTACTTCTTGATCCCTCTAAGGGAATGAGTATACCTTAATTATCTACTGAGCA
TGATAAGGTAAGGTACGTGCAGATTCGTATTAGGAGGTCATGATTTTCTTCAATTTA
GGTCTATGTTTTAAAAGGGGATTTGTTTAAAAAGATTTATTTATTATGTATACAATGT
TCTGCCTGGAGAGAGAGAGAGAGAGACAGAGACAGAGACAGAGACAGACAGACA
CACCGACTGACTGACTGACTAAGTGAGAATGGGAGAATGGTGTGGACTTTTTTCAA
CCTCAAAGCTCACCCCAGAAGAGGGCCAGAAGAGAGCACAAGATCTCATTATAGAT
GGATACGAGCCACTGTGTGGTTGCTGGGAATTGAACTCAGGACCTCTGGAAGAATA
GCCAATGTTCTTAACCTCTGAACTATCTCCAGCCCAAAGGGGGAAATTTGATTCTC
AACGAATTTCCATATATATATATATATATATATATATTTTTAAAGATTTATTTATTATTATA
TGTAAGTACACTGTAGCTGTCTTCAGACACTCCAGAAGAGGGAGTCAGATCTTGTT
ACGGATGGTTATGAGCCACCATGTGGTTGCTGGGATTTGAACTCTGGACCTTCGGA
AGAGCAGTCAGGTGCTCTTACCCACTGAGCCATCTCACCAGCGCCCCCCCCCCCC
CCCGTATATATTTTTAAATGAGTCTTAGTTTTTTGAGTTTTTTGAGTCAGCACTAGCA
AACATTCGGGTGATTTCAATTTTTCTTCTCCCAAATCTACAATGGAAGTGCTATGAC
CCTACAGGTATTCTCATATTTCATTGTGTTTGTTGGCAGCCGAGCAGAGATTCCCAT
GCCTAACAGTGGTTTTCCTTATGGTATTACCAAGCTTCAGTTTCTTTTTTCTTTTTAA
AATTGGTTTATTTATTTTTTTATTTTATGTGCACTGTTTTGCCTGCATGGATGTCTCT
AAGAGAATGTTGGATCCTCTGGAACTGGAGTTAGAGATAGTTGTGAGCTGCCATGT
AGATGCTGGGAATTGAATCCCGGGTTCTCTGAAAGAGCATCTAATGCTCTTGACTG
CCAGCCTTCTCTCCTGCCCCCAACTTAAGTTCTTTTTTCTTTTTCTTTTTCTTTTCCT
TTTCCTTTTGGTTTTTTGTTTTTTGTTTTTTTGAGACAGGGTTTCTCTGTGTAGCTC
TGGCTGTCCTGGAACTCACTTTGTAGACCAGGCTGGCCTCGAACTCAGAAATCCAC
CTGCCTCTGCCTTCCGAGTGCTGGGATTAAAGGCGTGTGCTACCACGCCTGGCCCC
AACTTAAGTTCTTAATGAAATACTTTTTATCATTTCTGAAGTGGTTAGAAAAGATTAA
TTGTATGTTAATTTACAATTTAACTTATTTCTAATAAATATTTCAAAGTATGTTTCAAT

TAAGGGCTATTCAGATGGATCTTGAATAAATCACAAGTTGATTTTATTATTACTGATA
ACAGTTAATTTTTTTTTTCATTTTTTTAAAAAGATTTATTTATTTTATCTATGAGTACG
CTGTTGCTGTCCTCTGACACACCAGAAGAGGGCATCAGATCCCATTATAGATGGTT
GTGAGCCACCATGTGGTTGCTGGGAATTGAACTCAGGACCTCTGGAAGAGCACTT
AGTGATCTTAACCACTGAGCCATCTCTCCAGCCCTTTTTCTTTCTATTTTTTTTAAA
GATTTATTTATAATTATACATAAGTACACTGTAGCTGTCTTCAGATGCACCAGAAGA
GGGCGTCAGATCTTATTAGGGGTGGTTGTGAGCCACCTTGTGGTTGCTGGGATTTG
AACTCAGGACCTTTGGAAGAGCAGTCACTGAGTGCTCTTACCTGCTGAGCCATCTC
ACCAGCCCTCTCCAGCCCTTTTTCATTTTTAAATATATTCTTTTATGCCTGTTTGGAG
AGAAATGAGTGTCTAAACAAGGATGTTAAAGGTGTTTGTTAGATGAGAAGAGTGTA
GTGATAGAGTCGTAAGCTGGAAGGACCTGACAAGGATCACAGAGATATTTGTAGGT
GGTGGATCTGTGTGTGTGGCTCAGAGGAGAGTGATACTGCCTTCTCTTTTCTTTAT
TAGCTGTTATTTAGCTTCTGGGTCAAGTGGTAGAGATCTGACACTCAGTTCTCACTT
TCCCTTACAGACGGGCTTGTCTGTAGTCACCATTGTGAATGTGAGTGAGTAGTGGG
TTTCACACTTGTGTGTGGTGGGTGCCAGTCTTTTCTCCTAGGAAATGAAGGCAGTT
TATTCTCTTGTTTTCAAACCTTAGGAATTCCTAACCATTAGAGCTTTTAAAGTGGTA
ATGCTGTTCTGTTGCTTGTTACGCATTGTTCTAATTACAGTGGCTTTAAATGCAGGC
TCAGAATTTCTCAAAACCCTGTTTTCCAGATAATGCTTATGATCCTGATGTGAATGC
TAAACAGATCTGGATCGATAAAACAGTGATTAGTGACCATATATGCTTGACATTCAC
TGATAATGGCAACGGGATGACAGCAGATAAGTTACATAAAATGCTAAGGTAAAAAC
GTGTTACAGTTTGGGGAATGATGACTTCTGTCTTGAAAATTATCCTGTGTCATGCAA
AAAACCATTTCATAGCCTTAAGCTTTGTCTTTAAAACCTGAAATGCTATGCCTACAG
TGTGGTTAGGTCTTCTGTTATTTTCTACAGCCAGGCTATGCACACCACTTCATATCC
CCACCATGCTCAGCATCCCTGCCCATATCTGCCTTACTTAAAATTCCAGCATCCCTT
TGGGATTGTATAATAGCTATTTTTACTTGGTATAAATTAGACTTAACTTGTGTTTCTA
ATGGGATTTACTATTGGAGATGGGGAGTTGTCTTTAAAGAGACTGAAGAGGCATAA
AATAGTCAGGTGTGTCTGCAGTGGGTGCTGAAGTGTACAGTAAAGCAGAGGGAGA
GTCTAGATGCAGAAGCCTTCAGTTGTCTCCTAAATGTCTACCGGGTAGGGGAGGCG
CTTTTCCCAAGGCAGTCTGGAGCATGCGCTTTAGCAGCCCCGCTGGGCACTTGGC
GCTACACAAGTGGCCTCTGGCCTCGCACACATTCCACATCCACCGGTAGGCGCCAA
CCGGCTCCGTTCTTTGGTGGCCCCTTCGCGCCACCTTCTACTCCTCCCCTAGTCAG
GAAGTTCCCCCCCGCCCCGCAGCTCGCGTCGTGCAGGACGTGACAAATGGAAGTA
GCACGTCTCACTAGTCTCGTGCAGATGGACAGCACCGCTGAGCAATGGAAGCGGG

TAGGCCTTTGGGGCAGCGGCCAATAGCAGCTTTGCTCCTTCGCTTTCTGGGCTCAG
AGGCTGGGAAGGGGTGGGTCCGGGGGCGGGCTCAGGGGCGGGCTCAGGGGCGG
GGCGGGCGCCCGAAGGTCCTCCGGAGGCCCGGCATTCTGCACGCTTCAAAAGCGC
ACGTCTGCCGCGCTGTTCTCCTCTTCCTCATCTCCGGGCCTTTCGACCTGCAGGTC
CTCGCCATGGATCCTGATGATGTTGTTGATTCTTCTAAATCTTTTGTGATGGAAAAC
TTTTCTTCGTACCACGGGACTAAACCTGGTTATGTAGATTCCATTCAAAAAGGTATA
CAAAAGCCAAAATCTGGTACACAAGGAAATTATGACGATGATTGGAAAGGGTTTTA
TAGTACCGACAATAAATACGACGCTGCGGGATACTCTGTAGATAATGAAAACCCGC
TCTCTGGAAAAGCTGGAGGCGTGGTCAAAGTGACGTATCCAGGACTGACGAAGGT
TCTCGCACTAAAAGTGGATAATGCCGAAACTATTAAGAAAGAGTTAGGTTTAAGTCT
CACTGAACCGTTGATGGAGCAAGTCGGAACGGAAGAGTTTATCAAAAGGTTCGGT
GATGGTGCTTCGCGTGTAGTGCTCAGCCTTCCCTTCGCTGAGGGGAGTTCTAGCGT
TGAATATATTAATAACTGGGAACAGGCGAAAGCGTTAAGCGTAGAACTTGAGATTAA
TTTTGAAACCCGTGGAAAACGTGGCCAAGATGCGATGTATGAGTATATGGCTCAAG
CCTGTGCAGGAAATCGTGTCAGGCGATCTCTTTGTGAAGGAACCTTACTTCTGTGG
TGTGACATAATTGGACAAACTACCTACAGAGATTTAAAGCTCTAAGGTAAATATAAA
ATTTTTAAGTGTATAATGTGTTAAACTACTGATTCTAATTGTTTGTGTATTTTAGATT
CCAACCTATGGAACTGATGAATGGGAGCAGTGGTGGAATGCAGATCCACTAGAGTC
GATCGACTAGAGCTGGTACCGCGGCCATTACCCTGTTATCCCTAGGCCGCCACCGC
GGTGGGCTTCCTCGCTCACTGACTCGCTGCGCTCGGTCGTTCGGCTGCGGCGAGC
GGTATCAGCTCACTCAAAGGCGGTAATACGGTTATCCACAGAATCAGGGGATAACG
CAGGAAAGAACATGTGAGCAAAAGGCCAGCAAAAGGCCAGGAACCGTAAAAAGGC
CGCGTTGCTGGCGTTTTTCCATAGGCTCCGCCCCCCTGACGAGCATCACAAAAATC
GACGCTCAAGTCAGAGGTGGCGAAACCCGACAGGACTATAAAGATACCAGGCGTT
TCCCCCTGGAAGCTCCCTCGTGCGCTCTCCTGTTCCGACCCTGCCGCTTACCGGAT
ACCTGTCCGCCTTTCTCCCTTCGGGAAGCGTGGCGCTTTCTCATAGCTCACGCTGT
AGGTATCTCAGTTCGGTGTAGGTCGTTCGCTCCAAGCTGGGCTGTGTGCACGAACC
CCCCGTTCAGCCCGACCGCTGCGCCTTATCCGGTAACTATCGTCTTGAGTCCAACC
CGGTAAGACACGACTTATCGCCACTGGCAGCAGCCACTGGTAACAGGATTAGCAGA
GCGAGGTATGTAGGCGGTGCTACAGAGTTCTTGAAGTGGTGGCCTAACTACGGCTA
CACTAGAAGGACAGTATTTGGTATCTGCGCTCTGCTGAAGCCAGTTACCTTCGGAA
AAAGAGTTGGTAGCTCTTGATCCGGCAAACAAACCACCGCTGGTAGCGGTGGTTTT
TTTGTTTGCAAGCAGCAGATTACGCGCAGAAAAAAAGGATCTCAAGAAGATCCTTT

GATCTTTTCTACGGGGTCTGACGCTCAGTGGAACGAAAACTCACGTTAAGGGATTT
TGGTCATGAGATTATCAAAAAGGATCTTCACCTAGATCCTTTTAAATTAAAAATGAA
GTTTTAAATCAATCTAAAGTATATATGAGTAAACTTGGTCTGACAGTTACCAATGCTT
AATCAGTGAGGCACCTATCTCAGCGATCTGTCTATTTCGTTCATCCATAGTTGCCTG
ACTCCCCGTCGTGTAGATAACTACGATACGGGAGGGCTTACCATCTGGCCCCAGTG
CTGCAATGATACCGCGAGACCCACGCTCACCGGCTCCAGATTTATCAGCAATAAAC
CAGCCAGCCGGAAGGGCCGAGCGCAGAAGTGGTCCTGCAACTTTATCCGCCTCCA
TCCAGTCTATTAATTGTTGCCGGGAAGCTAGAGTAAGTAGTTCGCCAGTTAATAGTT
TGCGCAACGTTGTTGCCATTGCTACAGGCATCGTGGTGTCACGCTCGTCGTTTGGT
ATGGCTTCATTCAGCTCCGGTTCCCAACGATCAAGGCGAGTTACATGATCCCCCAT
GTTGTGCAAAAAAGCGGTTAGCTCCTTCGGTCCTCCGATCGTTGTCAGAAGTAAGT
TGGCCGCAGTGTTATCACTCATGGTTATGGCAGCACTGCATAATTCTCTTACTGTCA
TGCCATCCGTAAGATGCTTTTCTGTGACTGGTGAGTACTCAACCAAGTCATTCTGA
GAATAGTGTATGCGGCGACCGAGTTGCTCTTGCCCGGCGTCAATACGGGATAATAC
CGCGCCACATAGCAGAACTTTAAAAGTGCTCATCATTGGAAAACGTTCTTCGGGGC
GAAAACTCTCAAGGATCTTACCGCTGTTGAGATCCAGTTCGATGTAACCCACTCGT
GCACCCAACTGATCTTCAGCATCTTTTACTTTCACCAGCGTTTCTGGGTGAGCAAA
AACAGGAAGGCAAAATGCCGCAAAAAAGGGAATAAGGGCGACACGGAAATGTTGA
ATACTCATACTCTTCCTTTTTCAATATTATTGAAGCATTTATCAGGGTTATTGTCTCA
TGAGCGGATACATATTTGAATGTATTTAGAAAAATAAACAAATAGGGGTTCCGCGCA
CATTTCCCCGAAAAGTG

Supplementary Methods

HE staining

Eight-week-old testes and caudal epididymis of the *Morc3* homozygous mutant and wild type male mice were dissected and fixed in 4% paraformaldehyde for 2 h at 4°C. After washing in PBS containing 10% and 20% sucrose, they were embedded in OCT compound. The sections were stained with hematoxylin (Wako, Japan) and eosin. After staining, the sections were treated with ethanol and xylene. The image was obtained using the BZ-X710 microscope (Keyence, UK)

Immunoprecipitation, SDS-PAGE, and silver staining

The E16.5 or 3-week-old testes of wild type and the mutant mice carrying the

FLAG-NLS-ZFP and *FLAG-NLS-ZFP-MIWI2* transgene were homogenized in a lysis buffer (20 mM Tris [pH 7.5], 150 mM KCl, 0.5% TritonX, 2.5 mM $MgCl_2$, 1 mM DTT, RNasin (Promega), and a protease inhibitor tablet (Roche)). The lysates were freeze-thawed and treated with benzonase. The homogenates were centrifuged at 15000 × rpm for 10 min at 4°C and the supernatant was subjected to immunoprecipitation using anti-FLAG (FLA1; MBL) or MORC3 (D238-3; MBL, Japan) antibodies overnight at 4°C and protein G Dynabeads (Invitrogen, US) for 1.5 h at 4°C. The immune complex was washed with the lysis buffer three times. Immunoprecipitates were subjected to SDS-PAGE on a 7.5% gel. The gels were subjected to silver staining using the Silver Stain MS Kit (Wako, Japan) or western blotting.

The transfected 293T cells were treated with lysis buffer (20 mM HEPES [pH 7.5], 0.1% NP-40, 150 mM NaCl, 2.5 mM $MgCl_2$, 1 mM DTT, and protease inhibitor tablet (Roche)). The lysates were precleared with Protein G Dynabeads (Invitrogen, US) for 1 h at 4°C and incubated with anti-PA antibody (Wako, Japan), anti-FLAG (FLA1; MBL) antibody, or anti-MILI (PM044; MBL) antibody overnight at 4°C. The immune complex was washed with the lysis buffer three times. The immunoprecipitates were subjected to western blotting.

Isolation of sperm

Mouse sperm were isolated from the cauda epididymis of more than 10-week-old wild type (C57Bl/6) and the MORC3 mutant mice by dissecting tissue in PBS. Sperm were allowed to swim up for 30 min in 37°C with 5% CO_2. To avoid the contamination of somatic cells, the upper fraction was collected for bisulfite sequence analysis.

Northern blot analysis

Total RNA samples were prepared from testes using ISOGEN (Nippon Gene CO., LTD., Tokyo, Japan) and stained with 0.02% methylene blue. RNA Northern blot analysis was performed at 65°C in a solution containing 0.2 M $NaHPO_4$ (pH 7.2), 1 mM EDTA, 1% BSA, and 7% SDS. The membranes were washed with a 0.2 × SSC, 0.1% SDS solution at 65°C. The subcloned PCR products were labeled with [α-^{32}P]-dCTP and used as probes. The sequences used for the PCR primers were as follows: the 3′ noncoding region of IAP (GenBank accession no. X04120), nucleotides 4489–4793, and the 5′

noncoding region of type A LINE-1 (M13002), nucleotides 531–1642 and type TF LINE-1(D84391), nucleotides 874–1156.

Immunohistochemical staining

Testes of the *Morc3* homozygous and heterozygous mutant, wild type, and the *Mili* knockout male mice were dissected and fixed in 4% paraformaldehyde for 2 h at 4°C (for D30 after birth and 4-week-old testes), 2% paraformaldehyde overnight at 4°C (for 10-month-old testes), or 2% paraformaldehyde for 1 h at 4°C (for E14.5, 16.5, and 17.5 testes). After washing in PBS containing 10% and 20% sucrose, the testes were embedded in OCT compound. The cryosections blocked with 10% normal goat serum and 3% BSA in PBS for 0.5 h at room temperature were immunofluorescence stained, after treatment with HistoVT one (Nacalai Tesque Inc., Kyoto, Japan) for 4-week-old, 10-month-old, E16.5, and E17.5 testes. Sections were treated with anti-MORC3 antibody (D238-3; MBL, Japan), anti-MVH antibody (ab13840; abcam), anti-MILI antibody (#5940; Cell Signaling technology), anti-MIWI2 antibody (ab21869; abcam), anti-MIWI polyclonal antibody (#2079; Cell Signaling), or anti-LINE1 ORF1p antibody (ab216324; abcam) overnight at 4°C. Alexa Fluor 488- or 568-conjugated anti-rabbit immunoglobulins (H+L) or Alexa Fluor 488- or 568-conjugated anti-mouse immunoglobulins (H+L) (Molecular Probes, Eugene, OR, USA) were used as the secondary antibody for 1 h at room temperature. Nuclei were counterstained with 1 μg/mL DAPI. Immunostained cryosections were examined under a confocal microscope (LSM5Pascal, Carl Zeiss Co. Ltd).

Detection of small RNAs

The detection of small RNAs from E16.5 testes of the *Morc3* homozygous mutant and wild type (control) mice using anti-MIWI2 and MILI antibodies were performed as described previously [1].

ChIP-qPCR assay

The E16.5 testes were removed and treated with 1 mg/mL collagenase type II and 0.25% trypsin for 5 min at 37°C. The germ cells were treated with 1% PFA for 8 min at room temperature. After quenching of the PFA crosslinking reaction with 0.2 M glycine, the fixed cells were washed with PBS. The fixed cells were suspended in lysis buffer (50 mM

Tris pH 7.5, 140 mM NaCl, 10 µM EDTA, and 1% SDS) and sonicated to an average DNA fragment size of ~500 bps. After the addition of radio immunoprecipitation assay (RIPA) buffer (20 mM HEPES-NaOH [pH 7.5], 150 mM NaCl, 1 mM EDTA [pH 8], 1% NP-40, 0.5% deoxycholate, and 0.1% SDS), solubilized chromatin was clarified by centrifugation for 10 min at 15,000 × rpm at 4°C. The chromatin was incubated with anti-MORC3 antibody (D238-3; MBL, Japan) overnight at 4°C. Immune complexes were bound to Dynabeads protein G for 1.5 h at 4°C. The immune complexes bound to the beads were washed with RIPA buffer and Tris-EDTA. Immune complexes bound to protein G beads were suspended in elution buffer (20 mM Tris-HCl [pH 8], 300 mM NaCl, 1 mM EDTA, and 0.5% SDS) and incubated overnight at 65°C. After incubation, the samples were treated with 30 µg/mL RNase A for 1 h at 37°C, and 100 µg/mL proteinase K overnight at 56°C. DNA was extracted with phenol/chloroform and precipitated with ethanol and Dr. GenTLE (Takara Bio, Shiga, Japan) as a carrier. Precipitated DNA was resuspended in 50 µL water and analyzed by qPCR using the CFX384 Real-Time PCR system (BIO-RAD) with THUNDERBIRD SYBR qPCR MIX (TOYOBO, Osaka, Japan) and specific primers mentioned below.

Sperm morphology and motility

Sperm morphology was observed and sperm motility was analyzed as described previously [2].

Primer

(For Bisulfite sequencing)

Primers Sequence (5' – 3') are as follows;

H19 (GenBank acc. no. U19619), product size: 423 bp

1st (outside) Forward: GAGTATTTAGGAGGTATAAGAATT

1st (outside) Reverse: ATCAAAAACTAACATAAACCCCT

2nd (inside) Forward: GTAAGGAGATTATGTTTATTTTTGG

2nd (inside) Reverse: CCTCATTAATCCCATAACTAT

LINE-1(A) (GenBank acc. no. M13002), product size: ～310 bp

Forward: TTATTTTGATAGTAGAGTT Reverse: CAAACCAAACTCCTAACAA

LINE-1(TF) (GenBank acc. no. D84391), product size: ～270 bp

Forward: GTTAGAGAATTTGATAGTTTTTGGAATAGG

Reverse: CCAAAACAAAACCTTTCTCAAACACTATAT

IAP, product size: 259 bp (ch3)

1st Forward: TAAGATAAAAGTTTGTAATGGTGGGAGAT

1st Reverse: ATTCTAAAATAAAATATCCCTCCTA

2nd Forward: AAATAAATTGTGGGAAGT

2nd Reverse: CAAAAAAAACACCACAAACCAAAAT

(For RT-qPCR)

Primers Sequence (5' – 3') are as follows;

Chr 7(1)-1 Forward : AGGGGGCTGTAATGAGTTCC

Reverse : AACATCACGAGGTCCAAAGC

Chr 7(1)-2 Forward : AGCTGAAAGTGCCAGGTGCCTTC

Reverse : TAGAGCTCTATTGAGTTGAGATC

Chr 7(1)-3 Forward : CAAGGCATTGGTTTGCCACGTG

Reverse : ATGGCAAAACTAACTGAGGTG

Chr 7(1)-4 Forward : TGAGCATCCTTTCCCTCCTGGA

Reverse : CAAGACAAGCAGACAGATATG

Chr 7(1)-5 Forward : CAGGCATATGGCCATAGAGTA

Reverse : GAGAAGAGGGAACCTATACTGA

Chr 10-1 Forward : AGCGACAGGTTTTCAGCGCTTC

Reverse : CTGCCCCGCACACAAGAGATC

Chr 10-2 Forward : CAGAGAGTTAGTGCAACGCTG

Reverse : ATTCAGCAGAAGGTTCTCCAG

Chr 10-3 Forward : ATGAAGGTGGCAGAAACTCCA

Reverse : ACTGGCAACTCTTTTTGCCCTG

Chr 10-4 Forward : AGGGCAAAAAGAGTTGCCAGTC

Reverse : ATCGAGGCAAAACCTCTAGCA

Chr 10-5 Forward : AGACACGTGCAACTCTTACTT

Reverse : CGTGCATGGTATTTGCAGCTG

Chr 10-6 Forward : CCAGGTCTTCCTAGCACATC

Reverse : GACTCCCCTCCTGTGTGTGTG

Chr 8-1 Forward : AGTTCCTTTCAGGGGTTTTCAC

Reverse : TGCACAGAGAAAAGCTGGGTG

Chr 8-2 Forward : CAGCCAGATGCAGATTTATTTTT

Reverse : GCACATGGGTGAAGAAAGGT

Chr 7(2)-1 Forward : ACTATAGCCTAGCTTAGTGTG

Reverse : TCTGGGGTAGCTGACAGTGATC

Chr 7(2)-2 Forward : AACAGACTACAACCACTACTG

Reverse : CTATAAATTACTCAAGCAGGGTG

β-actin Forward : CGGTTCCGATGCCCTGAGGCTCTT

Reverse : CGTCACACTTCATGATGGAATTGA

(For ChIP-qPCR)

Chr 7(1) promoter Forward : ACTCACCTGGCTCTCTGAAGGTTC

Reverse : GTTGCCCTAGTGCGCATGCGT

(For genotyping)

(For MORC3 (ΔFRT) Detection) Primers Sequence (5' – 3') are as follows;

Forward : AGTTGCTTTCAGGTGGATTAC   Reverse : TGCAATACCACCTGAAAGATG

(For MORC3 (ΔloxP) Detection) Primers Sequence (5' – 3') are as follows;

Forward : GTATGCCACCAAATGTTCAAG   Reverse : TGCAATACCACCTGAAAGATG

(For *Tnap-Cre* transgene Detection) Primers Sequence (5' – 3') are as follows;

Forward : GCATTACCGGTCGATGCAACGAGTGATGAG

Reverse : GAGTGAACGAACCTGGTCGAAATCAGTGCG

Reference

1 Kuramochi-Miyagawa, S. *et al.* DNA methylation of retrotransposon genes is regulated by Piwi family members MILI and MIWI2 in murine fetal testes. *Genes & development* **22**, 908-917, doi:10.1101/gad.1640708 (2008).

2 Miyata, H., Abbasi, F., Visconti, P. E. & Ikawa, M. CRISPR/CAS9-mediated amino acid substitution reveals phosphorylation residues of RSPH6A are not essential for male fertility in micedagger. *Biol Reprod* **103**, 912-914, doi:10.1093/biolre/ioaa161 (2020).

Supplementary Figure and Item legends

Supplementary Figure S1

MORC3 as an interaction partner of MIWI2

(a) Full-length gel of Figure 1a.

(b) Full-length blots of Figure 1b.

(c) Full-length blots of Figure 1c.

(d-i) Full-length blots of Figure 1d.

(j) *In vitro* co-IP assays for MILI and MORC3. HEK 293T cells were transfected with the plasmids expressing Myc-His-tagged MILI and Flag-tagged MORC3. The 293T cells were co-transfected with the tagged protein expression constructs. The lysates were immunoprecipitated with the anti-MILI or anti-Flag antibodies and subsequently subjected to western blotting with these antibodies. Each of IP : MILI, IP : Flag, and input samples was run on the same gel and transferred. Uncropped blots were shown in Supplementary Figure S1k.

(k) Full-length blots of Supplementary Figure S1j.

(l) Co-immunostaining of the testes of the E17.5 wild type mice with the anti-MORC3 antibody (red), anti-MIWI2 antibody (green), and DAPI (blue) for DNA are shown. Scale bar, 20 μm.

Supplementary Figure S2

Conditional gene-targeted disruption of *Morc3* by *Cre-loxP* system

(a) PCR genotyping of 12-day-old *Morc3* gene-targeted male germ cells purified by the anti-EpCAM antibody.

(b) Co-immunostaining of the testes of the E14.5 wild type and MORC3-cKO mice with the anti-MORC3 antibody (red), anti-MVH antibody (green), and DAPI (blue) for DNA are shown. Scale bar, 50 μm.

(c) Co-immunostaining of the testes of the 30-day-old MORC3-heterozygous and the mutant mice with the anti-MORC3 antibody (red), anti-MIWI antibody (green), and DAPI (blue) for DNA are shown. Scale bar, 100 μm.

(d) Spermatozoa from the cauda epididymis.

(e) Percentage of motile spermatozoa. No significant differences were found (by *t*-test). N=3. Error bars denote standard deviation (SD).

(f) Velocity parameters of spermatozoa. There were no significant differences in all the parameters (by *t*-test). N=3. VAP = average path velocity, VSL = straight line velocity, VCL = curvilinear velocity. Error bars denote standard deviation (SD).

(g) The results of the mating test using wild type female mice coupled with MORC3-cKO or wild type adult male mice. The number of plug and delivery in using each MORC3-cKO adult male mouse is described. Litter size is the number of pups in each delivery.

Supplementary Figure S3

Supplementary figures of uncropped northern blots and co-immunostaining of the testes of the E17.5 MORC3-heterozygous and the mutant mice.

(a-d) Northern blots featured in Figure 3a.

(e) Co-immunostaining of the testes of the E17.5 MORC3-heterozygous and the mutant mice with the anti-MORC3 antibody (red), anti-LINE1-ORF1p antibody (green), and DAPI (blue) for DNA are shown. Scale bar, 20 µm.

Supplementary Figure S4

Small RNAs in the embryonic MORC3 mutant testes

(a) Expression of micro RNAs (miRNAs) in the wild type and MORC3 mutant embryonic testes.

(b and c) Western blotting analysis of the immunoprecipitated proteins using the anti-MIWI2 and -MILI antibodies from testis lysates of E16.5 wild type and MORC3-cKO mice. Each of IP : MIWI2 and IP : MILI samples was run on the same gel and transferred (b).The samples of IP : MIWI2, IP : MILI, and input were run on the same gel and transferred (c). Uncropped western blots were shown at bottom. The immunoprecipitated RNAs were used for radiolabeled piRNA assay in Figure 5a (b) and deep-sequencing analysis (c).

(d) Full-length images of Figure 5a.

(e) Length distribution of MIWI2- and MILI-associated 25–31 nt small RNAs derived from each strand (sense or antisense) in E16.5 wild type and MORC3-cKO testes.

(f) Nucleotide distribution of the 1st or 10th nucleotide of piRNAs from each strand in MIWI2- and MILI-associated piRNA libraries from wild type and MORC3-cKO testes.

(g) Co-immunostaining of the testes of the E16.5 MORC3-heterozygous and the mutant

mice with anti-MIWI2 antibody (red), anti-MORC3 antibody (green), and DAPI (blue) for DNA are shown. The nucleus is surrounded by a white line. Scale bar, 20 μm.

(h) Co-immunostaining of the testes of the E16.5 MORC3-heterozygous and the mutant mice with anti-MILI antibody (red), anti-MORC3 antibody (green), and DAPI (blue) for DNA are shown. Scale bar, 20 μm.

Supplementary Figure S5

Expression of piRNA precursors from representative embryonic piRNA clusters

(a and b) Structure of the piRNA clusters (Chr 8 and Chr 7 (2)) and positions of individual primer set (upper). The region of IAP sequence in Chr 7 (2) cluster is described with red bars. Quantitative RT-PCR for the expression analysis of piRNA precursors transcribed from embryonic piRNA clusters using E16.5 wild type and MORC3-cKO embryonic testes (bottom). Data is normalized to β-actin and is shown as means and SD (Error bar) from more than triplicate PCR reactions. Significant differences (*$p < 0.05$ by the *t* test) between wild type and MORC3-cKO data using primer sets for the Chr 8-1 and 2 positions are shown (a).

(c) ChIP-qPCR analysis of E16.5 wild type embryonic testis with anti-MORC3 antibody. The embryonic testis lysates were chromatin-immuno-precipitated with control IgG and anti-MORC3 antibody. N=3. The promoter region of Chr 7 (1) piRNA cluster was analyzed. Error bars denote standard deviation (SD). The asterisk shows statistically significant differences (p<0.01 by *t*-test).

Supplementary Figure S6

A gene-targeting construct for *Morc3*

Exons are shown as filled boxes and *loxP* and *FRT* sites as empty arrowheads and black arrows. The resultant allele lacks exon 2 (containing a part of Histidine kinase/HSP90-like ATPase superfamily domain).

Supplementary Item S1

The DNA sequence of the targeting vector (pDTMorc3neo)

The sequences of the vector (pDT), loxP, exon 2, and FRT are shown in red, blue, green, and pink, respectively.
